# A wireless millimetric magnetoelectric implant for the endovascular stimulation of peripheral nerves

**DOI:** 10.1038/s41551-022-00873-7

**Published:** 2022-03-31

**Authors:** Joshua C. Chen, Peter Kan, Zhanghao Yu, Fatima Alrashdan, Roberto Garcia, Amanda Singer, C. S. Edwin Lai, Ben Avants, Scott Crosby, Zhongxi Li, Boshuo Wang, Michelle M. Felicella, Ariadna Robledo, Angel V. Peterchev, Stefan M. Goetz, Jeffrey D. Hartgerink, Sunil A. Sheth, Kaiyuan Yang, Jacob T. Robinson

**Affiliations:** 1 Department of Bioengineering, Rice University, Houston, TX USA; 2 Department of Neurosurgery, University of Texas Medical Branch, 301 University Blvd, Galveston, TX USA; 3 Department of Electrical and Computer Engineering, Rice University, Houston, TX USA; 4 Applied Physics Program, Rice University, Houston, TX USA; 5 Neuromonitoring Associates, LLC, Las Vegas, NV USA; 6 Department of Electrical and Computer Engineering, Duke University, Durham, NC USA; 7 Department of Psychiatry and Behavior Sciences, School of Medicine, Duke University, Durham, NC USA; 8 Department of Pathology, University of Texas Medical Branch, 301 University Blvd, Galveston, TX USA; 9 Department of Neurosurgery, School of Medicine, Duke University, Durham, NC USA; 10 Department of Biomedical Engineering, Duke University, Durham, NC USA; 11 Department of Engineering, University of Cambridge, Cambridge, UK; 12 Department of Chemistry, Rice University, Houston, TX USA; 13 Department of Neurology, UTHealth McGovern Medical School, Houston, TX USA; 14 Department of Neuroscience, Baylor College of Medicine, Houston, TX USA

**Keywords:** Biomedical engineering, Peripheral nervous system, Neurological disorders, Electrical and electronic engineering, Neuro-vascular interactions

## Abstract

Implantable bioelectronic devices for the simulation of peripheral nerves could be used to treat disorders that are resistant to traditional pharmacological therapies. However, for many nerve targets, this requires invasive surgeries and the implantation of bulky devices (about a few centimetres in at least one dimension). Here we report the design and in vivo proof-of-concept testing of an endovascular wireless and battery-free millimetric implant for the stimulation of specific peripheral nerves that are difficult to reach via traditional surgeries. The device can be delivered through a percutaneous catheter and leverages magnetoelectric materials to receive data and power through tissue via a digitally programmable 1 mm × 0.8 mm system-on-a-chip. Implantation of the device directly on top of the sciatic nerve in rats and near a femoral artery in pigs (with a stimulation lead introduced into a blood vessel through a catheter) allowed for wireless stimulation of the animals’ sciatic and femoral nerves. Minimally invasive magnetoelectric implants may allow for the stimulation of nerves without the need for open surgery or the implantation of battery-powered pulse generators.

## Abstract (web summary)

An endovascular wireless and battery-free millimetric implant enables the stimulation of peripheral nerves that are difficult to reach via traditional surgeries.

## Main

Bioelectronic modulation of neural activity is a powerful tool for treating many disorders, especially when these disorders cannot be effectively managed with conventional therapies. For example, electronic devices that stimulate neural activity are effective for treating disorders such as Parkinson’s Disease, epilepsy, chronic pain, hearing loss and paralysis^1–7^. These devices are most effective when implanted in the body where they can selectively stimulate the desired nerve targets; however, the invasiveness of the implantation can introduce additional risk for the patient. Invasive implants can also lead to complications such as chronic inflammation, which can further degrade device functionality and lead to failure^8–10^.

The vascular system that accompanies nerves as part of the neurovascular bundle provides a less invasive route for approaching nerve targets^11^. Existing neural implants for nerve targets such as the dorsal root ganglion (DRG) can suffer from site infection that results in device explantation and follow-up surgeries^12^. Millimetre-sized endovascular neural stimulators (EVNS) delivered via an intravascular catheter to deep tissue targets with a minimally invasive procedure through the blood vessels within the body would leave the target tissue undisturbed. As a result, endovascular deployment of devices is often associated with lower risk compared with open surgical approaches: recovery times are drastically reduced and site infections are extremely uncommon^11^. Given these advantages, an endovascular approach to neural stimulation would be attractive for the multitude of central and peripheral nerve targets that are adjacent to vascular structures, such as targets in the deep brain, peripheral nerves and the heart^13–15^. Recently, several new endovascular bioelectronic devices that exemplify the benefits of stimulating neural tissue through the vasculature have been developed^16–19^. However, these devices have stimulation leads that are wired to pulse generators or centimetre-sized inductive coils. The long lead wires and implantation of centimetre-sized devices create additional failure points and require an open surgery that reduces some of the benefits of an endovascular surgical approach^20^.

By miniaturizing the bioelectronic implants to a diameter of a few millimetres, it would be possible to deliver endovascular neuromodulation therapies entirely with minimally invasive procedures that rely on percutaneous catheters. To sufficiently miniaturize the device to the size constraints of the catheter (<3 mm diameter), some form of wireless power is necessary to replace the bulkier batteries if we expect long-term operation. While several innovative wireless power transfer modalities have been demonstrated, including far-field radio frequency radiation, near-field inductive coupling, mid-field electromagnetics with hybrid inductive and radiative modes, ultrasound and light, there has yet to be a demonstration of a mm-sized wireless and digitally programmable neural stimulator that operates at a depth of several centimetres in a large animal model^21–36^.

Here we turn to magnetoelectrics (ME) as a wireless data and power transfer technology due to its large power densities, high tolerance for misalignment and ability to operate in deep tissue when compared with alternative wireless power technologies for bioelectronic implants^37,38^.

Our results show that it is possible to safely stimulate peripheral nerves using electrodes placed inside the blood vessels, and that we can deliver the stimulation using a mm-sized bioelectronic implant. By combining ME data and power delivery with a custom application-specific integrated circuit (ASIC), we achieve a miniature device that is only 3 × 2.15 × 14.8 mm³ when fully encapsulated. Compared with miniature ultrasound-powered devices, our MagnetoElectric-powered Bio ImplanT (ME-BIT) maintains functional power levels over a larger range of translational and angular misalignment, and does not need ultrasound gels or foams to couple energy from the transmitter^29,31–33^. Furthermore, in comparison with previous in vivo demonstrations of ME-powered devices that were not digitally programmable^37^, the ME-BIT technology described here can receive digital data via the ME effect to programme the amplitude and timing of the electrical stimulus. As proof-of-concept, we show that these ME-BITs can be powered several centimetres below the tissue surface and can electrically stimulate peripheral nerve targets through the vasculature in a large animal model. These proof-of-principle studies open the door to minimally invasive bioelectronic therapies based on EVNS.

## Results

### ME combined with a custom ASIC enables a mm-sized neural stimulator

**Fig. 1:**
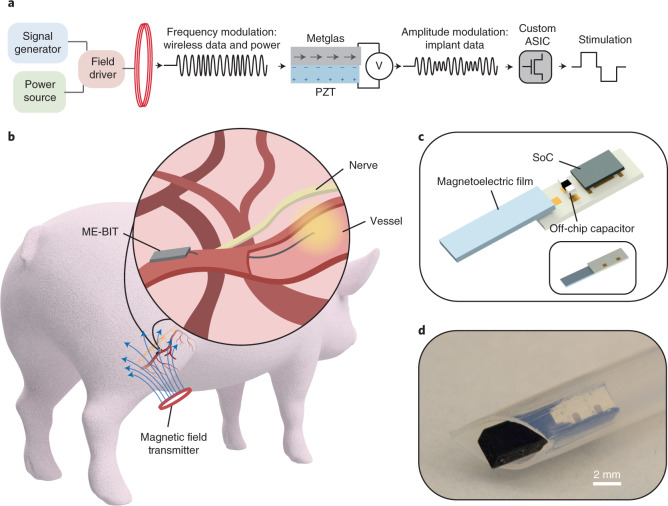
Overview of the wireless magnetoelectric endovascular neural stimulator. **a**, The overall EVNS system includes a magnetic field driver connected to a resonant coil that is tuned to the ME film resonant frequency. The coil outputs a frequency modulated magnetic field and switches between three different frequencies to modulate data and power received by the ME-BIT implant. The magnetic field is converted to an electric field by the magnetoelectric film. Specifically, the magnetostrictive layer Metglas mechanically deforms under the magnetic field and transfers the resulting strain to the piezoelectric layer PZT and induces a voltage. The amplitude of the voltage is then modulated by shifting the frequency of the applied field. The resulting voltage modulation received by the ME-BIT programmes the custom IC to output the desired stimulus waveform. **b**, For our proof-of-concept experiments, the ME-BIT is implanted proximally to a blood vessel deep within tissue and wirelessly powered through a magnetic coil in a pig. The implant’s stimulation lead is introduced into the vessel to stimulate nearby nerve targets. **c**, A rendering of the implant is shown with all the external components, including the system on a chip (SoC), external capacitor and the ME transducer. **d**, Photograph of the fully packaged device inside a 3D-printed capsule resting in a clear sheath. The implant is encapsulated with a non-conductive epoxy before being implanted into the body and has the potential to be delivered endovascularly.

To overcome the challenge of wireless data and power delivery to miniature bioelectronic implants, we developed a data and power delivery system on the basis of ME, which achieves high power densities within the safety limits for human exposure^39^. ME materials provide efficient power delivery for bioelectronic implants by directly converting magnetic fields to electric fields on the basis of the material’s properties^37,40^. In our case, we use a laminated bilayer material that consists of Metglas, a magnetostrictive layer, and lead zirconium titanate (PZT), a piezoelectric layer. When we apply a magnetic field to the material, the magnetostrictive material generates a strain that is coupled to the piezoelectric layer that, in turn, generates an electric field^37^. Thus, by applying an alternating magnetic field at the acoustic resonant frequency of the film, we can efficiently deliver power to our implant^37–39,41,42^. In addition to delivering power, we can also transmit data to our implant by modulating the frequency of the applied magnetic field. The frequency shift results in a change in the amplitude of the received voltage, which can be interpreted as a digital bit sequence that specifies the stimulation parameters for the implant^41,42^. Taken together, the complete wireless EVNS system consists of an external magnetic field transmitter, an ME film that harvests power and data from the magnetic field, and a custom integrated circuit (IC) that interprets the digital data and generates the electrical stimulus delivered by the electrodes (Fig. 1a). Figure 1b shows a conceptual overview of the system implemented in a large animal model where a surface coil can be used to wirelessly transmit a magnetic field to power and programme the implant for endovascular stimulation.

The ME-BIT itself consists of a magnetoelectric film with a size of 1.75 mm × 5 mm and a thickness of 0.3 mm for wireless power and data transfer, an ASIC for modulating the ME power and stimulation, and an external capacitor for energy storage (Fig. 1c), the system being packageable to fit within an 11 French catheter. For our experiments, we packaged the ME-BIT within a custom three-dimensionally (3D) printed polylactic acid capsule with on-board electrodes that can also be used to power external electrodes (Fig. 1d). With this design, the miniature capsule can not only be delivered through a minimally invasive catheter, but also serve as a complete neuromodulatory device that can receive power, undergo programming and transmit stimulation to neural tissue.

### A custom magnetic field transmitter enables data and power transfer at centimetre depths within safety limits

To deliver data and power to the implant, we designed a magnetic field transmitter that drives a high-frequency biphasic current into a resonant coil^41^. By maintaining transmitter power levels below 1 W, we can achieve field strengths >1 mT, sufficient to power the ME-BIT at depths of 4 cm within the safety limits.

**Fig. 2:**
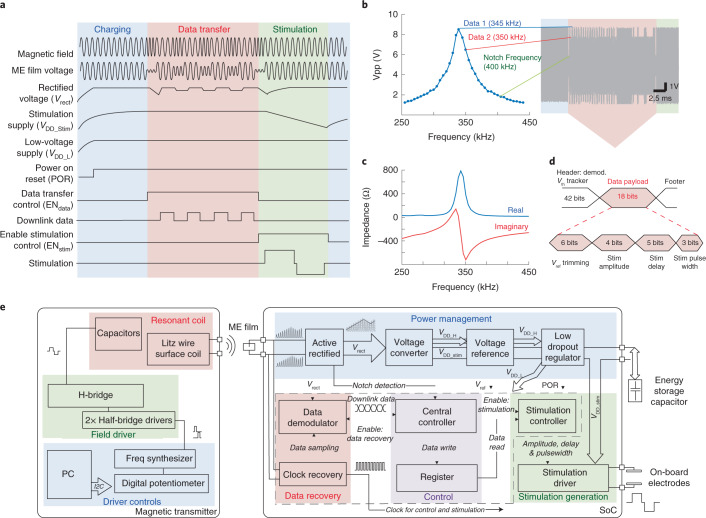
Timing and functional diagram for the ME-BIT. **a**, Timing diagram of the operation of the implant. The implant cycles through charging, data transfer and stimulating phase. The off-chip capacitor is charged in the charging phase to store energy for the stimulation. In data transfer, downlink data is received and demodulated by the ASIC to programme stimuli. The operation of the implant is fully controlled by the transmitter through frequency modulation, which changes the carrier frequency of the magnetic field for different induced voltages of the ME film. **b**, The peak-to-peak voltage for a film resonating in an extensional vibration mode at 345 kHz as a function of magnetic field frequency. Three different field frequencies are chosen for communication with the SoC. Data 1 indicates the highest voltage which is used for the charging phase and for encoding ‘data 1’. Shifting the frequency by 5 kHz results in a 25% drop in *V*pp that is used for ‘data 0’ as can be seen in the data packet highlighted in red. The third frequency or notch frequency is chosen at a > 50 kHz shift, which causes the *V*pp to drop > 85%. This is used to signify the beginning and end of the data transfer phase. **c**, The low film impedance at resonance lies at ~700 Ω to support milliwatt level power budgets. **d**, A summary of the data payload, including the header for demodulation threshold calibration and bits used for programming stimulation amplitude, delay and pulse width. **e**, Block diagram of the magnetic transmitter (left) and the custom ASIC of the implant (right). The magnetic transmitter contains a controller that interfaces with a personal computer (PC), a magnetic field driver and a resonant magnetic coil to generate a low-frequency alternating magnetic field. The ASIC interfaces with an ME film to wirelessly receive power and data, and consists of power management, data recovery, control and stimulation modules to drive programmable stimulation. Energy for the high-power stimulation is stored in the off-chip capacitor and stimulus is delivered through the on-board electrodes.

Because the amplitude of the ME voltage peaks at the acoustic resonant frequency, we can send digital signals to our ME-BIT by detuning the applied magnetic field frequency. Figure 2 shows our communication protocol with charging, data transfer and stimulation phases. As seen in Fig. 2b, we can select 3 frequencies to transmit digital data. The first frequency ‘Data 1’ corresponds to the mechanical resonance (345 kHz). This is the frequency of maximum voltage (and maximum power transfer), which we use as a digital 1, and for the charging and stimulation phases. The second frequency ‘Data 0’ is detuned by ~5 kHz. This frequency of 350 kHz produces a lower amplitude voltage, which is used as a digital 0. The third frequency is detuned by 55 kHz from the resonance peak and produces an even lower voltage than the ‘Data 0’ signal. This ‘notch frequency’ of 400 kHz is used to indicate the start of the data transfer and stimulation phases. By using the mechanical properties of the ME film to receive data on the basis of frequency modulation, we can avoid turning the transmitter coil on and off, which would require a settling time of 100 µs for our resonant transmitters. Given the fast settling time of this frequency modulation scheme, we find that 64 cycles of the carrier frequency can reliably transmit one bit, resulting in a 4.6 kbps data rate. We use a digital payload of 18 bits per stimulation, which accounts for a preamble and real-time calibration of the demodulation reference. This payload combined with the charging phase yields a maximum stimulation rate of 1 kHz, which is well within the range of typical neural stimulation applications^41^.

We estimate that this device can generate a maximum of 4 mW as long as the ME films can maintain a peak resonance voltage of >8 peak-to-peak voltage (Vpp) with a resistive source impedance lower than 1 kΩ (Fig. 2d). This power level is sufficient for many neural stimulation applications^43^.

### Custom IC provides digitally programmable stimulation with <9 µW power consumption

To deliver reliable stimulation independent of the coupling between the transmitter and the ME-BIT, the implant includes a custom ASIC that uses the digitally received data to programme the shape (monophasic or biphasic), the amplitude (0.3 V to 3.3 V with 4 bit resolution), the pulse width (0.05 ms to 1.2 ms with 3 bit resolution) and the delay (0.01 ms to 0.8 ms) of the stimulation. The stimulation reference voltage is also programmed by the downlink data to generate a stimulation supply voltage 10% higher than the desired amplitude. As a result, the implant achieves >90% stimulation efficiency *η*_stim_ for 1.5–3.3 V stimulation amplitude; when compared to the stimulation power (<9 mW), the power consumption of the SoC is negligible (<9 µW). Thus, we expect little heating due to energy loss on the chip. Furthermore, this high efficiency also reduces required transmitter power and its associated heating.

$${\eta}_{{{{\mathrm{stim}}}}} = \frac{{ < \mathrm{stimulation}}\,{\mathrm{voltage} > \times < \mathrm{stimulation}}\,{\mathrm{current} > }}{{ < \mathrm{stimulation}}\,{\mathrm{supply}}\,{\mathrm{voltage} > \times < \mathrm{stimulation}}\,{\mathrm{current} > }}$$

The ASIC, fabricated on 180 nm complementary metal-oxide-semiconductor (CMOS) technology (TSMC), measures only 1 mm × 0.8 mm while performing several functions to ensure robust stimulation and communication. The ME-induced alternating voltage is first rectified to the direct current (DC) voltage *V*_rect_ by a full-bridge active rectifier with an 84% voltage conversion efficiency. This rectified voltage is then converted by a DC voltage converter, which provides proper voltage and buffers energy on the off-chip capacitor Cstore for stimulation. The voltage converter also generates a high-voltage supply *V*_DD_H_ for other circuits for power management, including the low-dropout regulator and the voltage references generator, and guarantees cold startup of the system as well. A constant low-voltage supply *V*_DD_L_ of 1 V is provided by the low-dropout regulator for the controller, the data demodulator and the timing reference generator. To ensure proper system operation, a power-on-reset (POR) signal is triggered when *V*_DD_L_ stabilizes.

To maintain reliable functionality of implants under varying ME voltages caused by changes in transmitter–implant distance and alignment, the phase transitions of the IC are fully controlled by the transmitter through the short notches in ME voltage. In addition, the demodulation threshold for the amplitude-modulated data is generated autonomously at the beginning of the data transmission cycle to avoid data recovery errors due to changes in ME voltage. Meanwhile, a global system clock is extracted from the source by a low-power comparator-based clock recovery circuit, ensuring process and voltage invariant timing references for data sampling and stimulation.

### ME implant demonstrates high tolerance towards misalignment

**Fig. 3:**
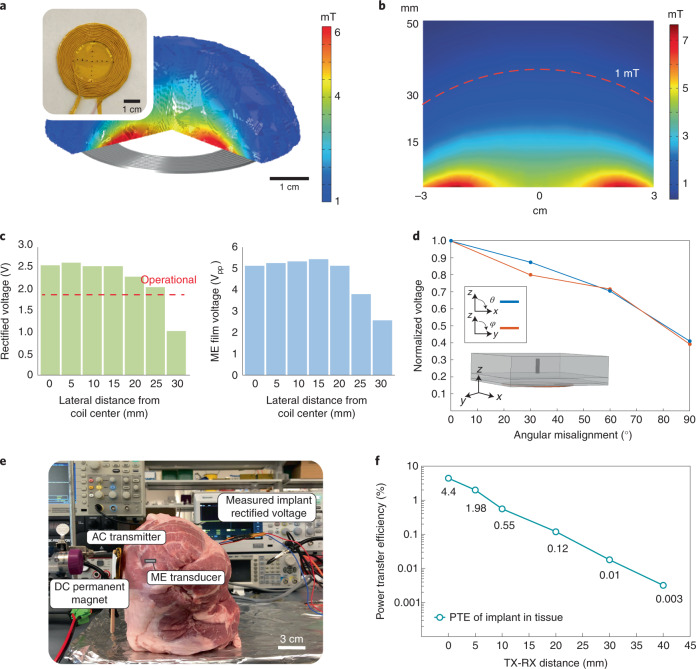
Characterization of ME power transfer. **a**, Finite element simulation of the magnetic field produced by one of the AC coil geometries used to power the ME implants. The coil shown is ~7 cm in diameter, made from 18 AWG litz wire and insulated with polyimide tape. The ME implant has been shown to maintain functional voltages at a field of 1 mT, and the COMSOL simulation demonstrates the space in which the implant remains functional where the edges are bound to be 1 mT. **b**, A 2D cross-section of the simulation from **b** is shown, where a 1 mT field can be generated at a distance of 30 mm and maintain lateral uniformity in case of translational misalignment (red dashed line). **c**, Experimental data measuring the implant’s rectified voltage (left) as the implant is moved away from the centre of the coil. For the device to remain functional, the rectified voltage needs to be >1.8 V as shown with the red dotted line. Also shown is the peak-to-peak ME voltage (right). **d**, COMSOL simulation of the ME-BIT placed within a layered block of tissue (20 mm muscle, 5 mm fat, 2 mm skin) is used to model angular misalignment tolerances in vivo for both *θ* and *φ* angular rotations. **e**, An experimental image is shown for an ex vivo model of porcine tissue. The magnetic field transmitter which is a combination of both the permanent magnet and AC coil, is placed on the left-hand side of the tissue. The encapsulated ME film powers an implant up to 40 mm within the heterogeneous tissue, while the rectified voltage of the implant is measured and used to calculate the power transfer efficiency. **f**, Measured PTE for the ME implant as a function of distance in tissue. At an operating distance of 30 mm, the transmitter power was ~6 W to maintain the 1.17 mW implant power, yielding a 0.01% efficiency.

We find that our magnetoelectric-based power transfer approach displays improved tolerance for translational and angular misalignment when compared with other mm-sized implants. Our simulations show that ME-BITs can tolerate approximately 3 cm translational misalignment from the centre of the transmitter coil and a depth of 3 cm in tissue. Using finite element modelling (COMSOL) to model the magnetic field generated by our 15-turn transmitter coil, we find an almost uniform magnetic field across 6 cm inner diameter of the coil (>70% of total transmitter area) as shown in Fig. 3a. The dashed line in Fig. 3b shows the boundary line of 1 mT, which is the operating field strength for the implant.

When we tested our 15-turn transmitter coil, we found that we could indeed power our ME films above our operating voltage (>3.6 Vpp) a distance of 3 cm in air from the surface of the coil, with a misalignment tolerance that matched the 6 cm inner diameter of the coil (Fig. 3c). This misalignment tolerance is more than 27 times greater than recently reported ultrasound-powered implants with a mm-scaled translational window^33^. The improved alignment tolerance would be advantageous for applications where an individual may want to align a transmitter several times a day or fit a wearable transmitter that may move and drift over time.

Furthermore, ME-based power transfer also demonstrates encouraging angular misalignment tolerances. In comparison with inductive coils that harvest power on the basis of magnetic flux, the ME materials harvest power on the basis of magnetic field strength. As a result, it has been shown that ME demonstrates more stable power transfer as a function of angular misalignment^39^. This angular stability is supplemented by the fact that the large magnetic permeability of the Metglas layer helps to concentrate the magnetic field lines along the length of the ME film^44^. To assess the angular tolerance of the ME-BIT in vivo, we used a COMSOL model to simulate how ME voltage is affected when it undergoes angular misalignment in tissue (see Methods). Because the simulated coil is radially symmetrical, we found that rotating the film in either the *θ* direction, as shown in Fig. 3d, or in the *φ* angular direction, resulted in the ME voltage decaying similarly with either angular change and being able to maintain >40% of the maximum voltage at a 90° rotation. Existing devices and implants that use ME antennas probably share similar angular tolerances and have been shown to be operational at large distances; however, these sub-mm devices primarily operate at much higher frequencies (60 MHz to 2.5 GHz)^45–47^. At these higher frequencies, tissue absorption and reflection become more substantial, which lowers the amplitude of the field that can be applied within the safety limits^48^. Furthermore, many of these demonstrations rely on the magnetic component of radiating electromagnetic waves, which is small compared to the electric field component. As a result, small ME devices that couple to radiating electromagnetic waves are used primarily for low-power sensing and communication applications rather than electrical stimulation, which requires more power.

### ME power is able to sustain operation of implant at centimetre distances within tissue

We found that the ME-BITs received enough power to function when implanted at centimetre depths in porcine tissue (Fig. 3e). Specifically, the ME film packaged inside the IC capsule was able to power the IC and maintain a sustained rectified voltage of 1.9 V. By adjusting the magnetic field while increasing the distance between the implant and transmitter coil, the ME-BIT was able to maintain a working voltage up to 4 cm (Supplementary Table 1). This energy was delivered through a ~3 mm air gap between the surface coil and tissue, demonstrating the ability to achieve non-contact wireless power transfer.

When characterizing the power coupling efficiency in tissue, we achieved functional power levels at a transmitter-receiver (TX-RX) distance of up to 4 cm, which is primarily limited by the size and power level of our magnetic field transmitter. At the surface of the coil, while the ME-BIT generated a peak power of 1.17 mW, the resulting peak efficiency of the implant was found to be 4.4% (Fig. 3f). To maintain a functional voltage on the implant at a depth of 4 cm, the coil current was increased from 0.23 A to 8.6 A at 0 mm and 40 mm distance, respectively (Supplementary Table 1 and Methods). While the maximum distance demonstrated here is 4 cm, ME voltage is primarily dependent on magnetic field strength, thus greater TX-RX distances may be achieved by optimizing driver electronics and transmitter designs.

### ME-BIT demonstrates programmability and fully untethered operation for direct nerve stimulation in rats

**Fig. 4:**
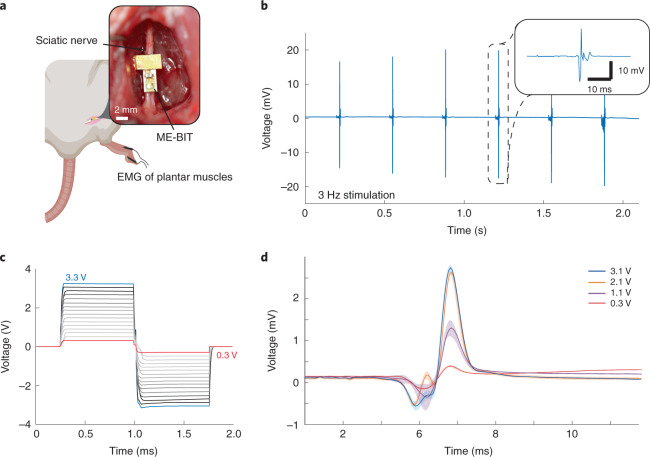
In vivo direct nerve stimulation in a rat model. **a**, Schematic of the implantation of a fully wireless ME-BIT in a rat model. The device is placed on top of the rat sciatic nerve and wirelessly powered from an external transmitter. The stimulating electrodes are two 1 mm × 1 mm gold pads spaced 2 mm apart on the bottom side of the circuit board. EMG recording electrodes are placed in the plantar muscles of the foot while a ground electrode is placed higher in the body. Also shown is an image of the actual device used for stimulation. **b**, The free-floating device is set to stimulate the nerve at 3 Hz with 3 V, 1.5 ms pulses and the recorded EMG signal is shown. The insert shows a close-up of the highlighted EMG trace. **c**, A graph showing programmed biphasic stimulus pulses of varying amplitudes. **d**, Averaged EMG recordings from the plantar muscles of the rat showing graded traces in response to varying levels of programmed stimulation powered by the wireless implant.

Proof-of-concept experiments show that wirelessly powered ME-BITs evoke repeatable compound muscle action potentials (CMAPs) along with observable leg kicks when placed in contact with the sciatic nerve. This miniaturized implant had a volume of 6.2 mm^3^ and weighed 30 mg, making this suitable for small rodent models, and was able to directly stimulate rat peripheral nerve (*n* = 2) in vivo (Fig. 4a). Stimulation for rat A, while the mote was fully untethered and powered at a distance of 1 cm, is shown in Fig. 4b, where a 3 V, 1.5 ms pulse width monophasic pulse train was applied at 3 Hz. Electromyography recordings of foot muscles showed waveforms that were time locked with the applied stimulus at the same frequency. Often necessary in neural interfaces, the stimulation parameters on the ME-BIT can be adjusted by sending the appropriate commands through the magnetic field. Not only is the implant able to adjust its stimulation amplitude from 0.3 V to 3.3 V as shown in Fig. 4c, it is also able to vary its pulse width and frequency to meet the demands of different neuromodulation applications and provide targeted therapies to account for variance from patient to patient. The programmability of the device is shown through an acute demonstration with rat B by varying the amplitude of the stimulus and observing the resulting graded EMG response. By adjusting the stimulation as well as the pulse width, the total charge delivered to the nerve could be controlled to directly affect the number of recruited motor units to elicit varying CMAP responses. In Fig. 4d, we innervated the sciatic nerve with monophasic pulses at 1 Hz while holding the pulse width at 1.5 ms and varying the amplitude from 300 mV to 3.1 V. The resulting CMAPs ranged in amplitude from 0.4 to 2.7 mV where the number of recruited muscle fibres seemed to saturate when increasing the stimulation amplitude from 2.1 to 3.1 V.

### The ME system demonstrates wireless endovascular nerve stimulation for multiple nerve targets

**Fig. 5:**
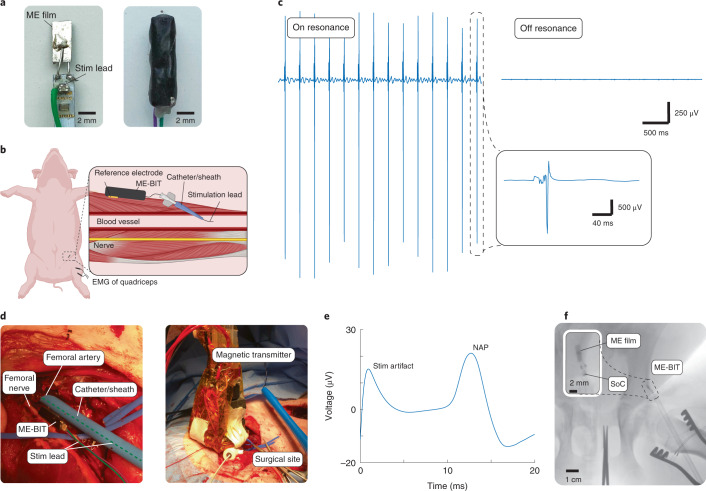
Endovascular peripheral nerve stimulation in a large animal model. **a**, Left: the exposed implant with ME film and soldered stimulation lead at the top of the PCB. Right: the fully encapsulated device in the 3D-printed box and covered in non-conductive epoxy. **b**, A schematic of the endovascular stimulation in the pig. The implant is placed near the targeted femoral artery and the stimulation lead is introduced into the vessel through a catheter. **c**, The resulting EMG is shown where recording electrodes are placed on the bicep femoris of the pig. The blue trace on the left shows monophasic stimulation at 10 Hz with the magnetic field on resonant, while the trace on the right is the control where the magnetic field and transmitter are tuned to be off-resonant. **d**, Left: close-up of the surgical site with a 9 Fr sheath entering the femoral artery, as well as the implant and femoral nerve. Right: the magnetic field transmitter on top of the skin, powering the intravascular device and stimulating the femoral nerve at a distance of ~1.5 cm inside the body. **e**, A time-averaged NAP was recorded with bipolar hook electrodes directly on the femoral nerve with wireless endovascular stimulation. **f**, An X-ray image of the ME implant endovascularly deployed from a 9 Fr sheath into the femoral artery. The ME film, capacitor and SoC can be seen in the X-ray image.

To demonstrate endovascular neural stimulation and the potential for clinical translation, we implanted the ME-BIT in a pig and demonstrated peripheral nerve stimulation from within the blood vessels using a wirelessly powered device. For this experiment, the film was mounted along the printed circuit board (PCB) and soldered to gold pads, with the stimulation lead wire soldered to an exposed pad on the top of the PCB before the device encapsulation (Fig. 5a). For the surgery, an incision was made in the hind leg of the pig to expose both the femoral nerve and the femoral artery. The ME implant was then placed into the surgical site and a 9 Fr sheath was then introduced into the femoral artery to allow access into the vessel. The parylene insulated stimulation wire connected to the implant was introduced into the vessel as shown in the schematic in Fig. 5b. Images of the surgical site shows the femoral nerve and a sheath entering the femoral artery with the encapsulated implant placed proximal to the vessel. The magnetic field transmitter was then brought to the surface of the skin to wirelessly power the implant at an implanted distance of 1.5 cm (Fig. 5d). By applying a 3 V monophasic stimulus pulse with 1.5 ms pulse width to the exposed tip of the endovascular wire, the device provided targeted monopolar stimulation with the reference electrode on the ME implant. As shown in Fig. 5c, we were able to stimulate the femoral nerve through the femoral artery at various stimulation frequencies including 10 Hz. Along with CMAPs, we recorded downstream nerve action potentials with bipolar hook electrodes shown in Fig. 5e, as well as time-averaged central somatosensory evoked potentials (SSEPs) (Supplementary Fig. 1). These recordings demonstrate that the stimulation was mediated by the nerves and is not a direct muscle stimulation. To rule out the possibility that our data could be explained by stimulation artifacts due to the applied magnetic field, we performed control experiments with the magnetic field detuned from the ME resonance wavelength (Fig. 5c). Although we transmitted the same communication protocol, because the magnetic field was detuned, the ME-BIT did not accurately receive the digital data and the implant did not deliver a stimulus.

We were also able to demonstrate EVNS of the intercostal nerves and the dorsal root ganglion, which are common targets for treating chronic pain^12^. As shown in Supplementary Fig. 2, we were able to introduce the insulated stimulation wire through the segmental artery to reach intercostal nerves and the DRG of the pig, which are both in direct contact with the intercostal arteries. Similar to femoral nerve stimulation, we applied a monophasic stimulus pulse at 3 V, 1.5 ms with varying frequencies from 1–10 Hz. The delivered exposed wire tip served as the monopolar electrode, while the return electrode was an on-board electrode located on the ME implant. We also were able to measure compound muscle action potentials that resulted from the stimulation and observed time-aligned muscle contractions in the chest wall. When we performed the same off-resonant frequency controls as we did for the femoral nerve stimulation, we found no response, supporting the fact that EVNS of the intercostal nerve was also a nerve stimulation from within the blood vessel.

The small mm-sized form factor of our ME-BITs also enables delivery of the entire device within the vasculature. As proof of concept, we deployed our ME-BIT through a 9 Fr sheath into the femoral artery as can be seen in Fig. 5f. The ME film and ASIC are visible through X-ray, which can allow for visualization and monitoring of the device post implantation. Tissue samples for histologic evaluation were also taken to assess for acute vascular damage from endovascular stimulation; no damage was observed as shown in Supplementary Figs. 3–5.

In summary, we demonstrate (1) that these miniature ME-BITs have sufficient power density to stimulate a clinically relevant large animal model from within a blood vessel and (2) the potential for wireless EVNS of multiple peripheral nerve targets. Because the implant can be deployed through a 9 Fr sheath (inner diameter of 3.09 mm), it would be possible to deliver the device using minimally invasive surgical procedures. For example, Fig. 5f shows an X-ray image of an endovascularly deployed ME-BIT in the femoral artery (see Methods). Utilizing the advantages of magnetoelectrics, the ME-BIT can be implanted deep within the tissue close to targeted areas without requiring lead wires that connect to a more superficial inductive coil.

## Discussion

This work shows a magnetoelectric-powered bioelectronic implant in a large animal model, and highlights several of the advantages of this wireless power-transfer technology for biomedical applications. Specifically, its large angle and lateral misalignment tolerance are favourable for the future use of wearable transmitters to power and communicate with the ME-BIT. Although the implant itself might only move a few millimetres once fixed within the tissue, it is easy to imagine misaligning a wearable transmitter by a centimetre or more, which remains within our alignment tolerances. Furthermore, the use of a wearable transmitter is also possible due to the low magnetic field strengths that are required to activate high voltages in these ME thin films. These thin films require only <1 mT field strengths for the power densities needed to activate neurons through stimulation by the material itself^37^ or by powering custom integrated circuits^41,42^. This will allow the technology to be readily translated into the clinic and may even permit patients to use implants at a home setting. Furthermore, because wireless power transfer scales favourably for ME in that power decreases linearly with implant size rather than a higher power as is the case with other wireless power technologies^37^, it may be possible to greatly reduce the size of the device to the point where it could fit in smaller vessels and be deployed to difficult-to-reach targets.

The data that we provide represents proof-of-concept evidence that peripheral nerves can be stimulated from within the blood vessels using a mm-sized wireless implant. Additional work is needed to develop this technology into a biomedical device for clinical use. For one, hermetically sealed packaging will be needed for chronic implantation of the device. Although thin-film packaging solutions have yet to be fully developed for clinical use, other wireless implants have shown that glass or ceramic casings can enable chronic operation^49^. Fortunately, the magnetic fields should easily penetrate these materials and thus they are not expected to degrade the power coupling efficiency. Long-term deployment of future endovascular bioelectronics may also require adjunctive therapies using blood thinning pharmaceuticals. Several factors that involve the implantation of devices within the vasculature can promote thrombosis; however, improved techniques along with antithrombotic regimens have been shown to decrease any catastrophic thrombosis due to stent implantations to <1%^50,51^. Furthermore, it has also been shown that extended implantation of cardiac pacing leads that develop occlusions can result in collateral venous channels that would reroute blood around the occlusion^52^. Future studies are needed to determine (1) how chronic deployment of the ME-BIT within the blood vessel could affect vasculature health and (2) the biocompatibility of the device, including the suitability of a hermetically sealed capsule for long-term implantation of the lead-containing PZT or other piezoelectric alternatives that do not contain lead, such as polyvinylidene fluoride^37^. Another safety concern for the long-term implantation of the ME-BIT are the interactions between the applied magnetic field and biological tissue. Our COMSOL simulations show that a field of 1 mT at an implant depth of 3 cm corresponds to a surface magnetic field of 7.7 mT, which results in an electric field and specific absorption rate that are within the IEEE safety limit of 101 V m^−1^ and 2 W kg^−1^ for unrestricted environments^36^. For other guidelines, such as those by the ICNIRP that have lower limits for magnetic field exposure, this device operates outside the compliance range^53^. Thus, future approval for these devices may depend on which standards are applied by the regulatory body. While we operate our device at an optimal rectified voltage of 2.5 V, the ME-BIT remains operational at voltages as low as 1.8 V, in which field strengths as low as 0.6–0.8 mT can still be used. Additionally, improvements to the ME materials that increase the power transfer efficiency (PTE) or reduced power consumption by the ASIC could allow these devices to operate with lower magnetic field strengths, which could make the devices compliant with additional safety standards.

As we miniaturize the implant and ME film sizes, we expect that the ME-BITs will still be able to function at centimetre depths in tissue. This is because the ME film voltage does not depend on the area of the film^37,43^. As a result, we expect that received power will only decrease linearly with the size of the film. The film voltage, on the other hand, is expected to remain constant, which will ensure that the voltages are large enough to operate the ASIC. Thus, we expect that the major effect of miniaturization would be longer charging times between stimulation pulses, which could decrease the maximum stimulation bandwidth. Future work must also address packaging and connectorization, which will probably need to be changed as devices approach sub-mm length scales. These efforts will be needed to compare ME-powered implants with other types of sub-mm-sized battery-free implants, and their compatibility with new minimally invasive delivery techniques which, although promising, have yet to demonstrate neural stimulation in a large animal model^54–56^.

Endovascular bioelectronics, such as the ME-BIT demonstrated here, opens the door for a wide variety of therapies that involve low-risk and high-precision implantable devices. Having bioelectronics implanted within the vasculature enables devices to be implanted in many parts of the body that are traditionally difficult to reach without having major risks of surgery. Additionally, bioelectronic implants with access to the bloodstream could enable real-time sensing of biochemicals, pH or oxygenation levels within the blood to provide diagnostics or support closed-loop-electronic medicine^57,58^. Overall, wirelessly powered mm-sized devices implanted within or near the vasculature could open up numerous opportunities for minimally invasive bioelectronic medicine.

## Methods

### Fabrication of ME-BITs

The external capacitor was mounted on the PCB before wirebonding the custom 0.8 mm × 1 mm IC to the board (Supplementary Fig. 6). After wirebonding, the die area was encapsulated with epoxy to maintain the structural integrity of the bonds. The magnetoelectric film was fabricated with a 127-µm-thick PZT (APC Int.) bonded to a 23-µm-thick layer of unannealed Metglas (2605SA1, Metglas) with a thin epoxy layer (Hardman Double/Bubble). The films were then laser cut by a femtosecond laser cutter to the desired shape to operate in the 300–400 kHz frequency range, at which the ME films operate in the fundamental extensional vibration mode. We chose to operate the ME-BIT in the extensional vibration mode as it has been shown previously with ME bilayer laminates that while the bending mode has higher magnetoelectric energy conversion efficiency, longitudinal resonance modes yield slightly higher voltage coefficients^59^. Future work can consider the usage of different resonant modes, including the primary bending mode at lower resonant frequencies. Depending on the iteration of the device, the geometry and strategy for interfacing the film with the board are slightly different. For the direct nerve stimulator, the film size was manufactured to be ~4 mm × 3 mm. This film was then coated with ~10 nm of titanium and ~40 nm of gold with radio frequency sputtering. The film’s non-coated side was bonded to an exposed pad on the PCB with conductive silver epoxy (Electron Microscopy Sciences) and allowed to cure at 60 °C for 20 min. The top electrical connection of the film was made by wirebonding the gold-coated side to the second exposed pad on the PCB. In the case of the endovascular device, the aspect ratio is more important as the device length is not as important as the width to fit inside a catheter/sheath and deployed in blood vessels. The films were cut out to be ~1.75 mm × 5 mm. Conductive silver epoxy was used to connect 30 AWG wire to the centre of the ME film. This ME film was then soldered directly to the two exposed pads on the PCB. The insulated stimulation wire was then soldered onto the top side of the PCB. A second wire shown in Figs. 5a and 5d (purple) was soldered to the reference electrode for more flexible positioning, but was ultimately not used in the experiment. The assembled device was then placed within a 3D-printed air-filled polylactic acid capsule, which allows the film to freely vibrate in air. The entire capsule was then sealed with non-conductive epoxy to provide more structural stability and prevent moisture from infiltrating the device. The assembled implant’s final dimensions are 3 × 2.15 × 14.8 mm³.

### Device testing and calibration

Before fully packaging with an enclosure for encapsulation, each device was checked through comprehensive functional tests. In addition to the pads connected to the film, the energy storage capacitor and the stimulating electrodes, the ASIC also has testing pads and readout circuits providing gateways to the internal signals, such as the verified voltage, the low-dropout regulator output and the demodulated downlink data. Through monitoring these signals at various conditions, such as different TX-RX distances and misalignments, we validated that the devices could operate properly in future implantation. The ASIC was designed with robustness against source amplitude changes and process variations. In addition, we calibrated some variables during the tests to further improve the reliability of device operation and the effectiveness of stimulation. First, the carrier frequency shift for amplitude modulation needs to be carefully set. Simply employing a large enough frequency change may ensure that the voltage difference between data ‘1’ and data ‘0’ is always sufficient (>100 mV), but would sacrifice the received voltage and power in the data transfer phase and hence suffer a smaller TX–implant distance. Therefore, an optimal frequency setting needs to be found to maximize the ME-induced voltage while still providing a large enough modulation index for correct data demodulation. Due to process variations in the manufacture of the ME laminate, this optimal frequency shift may demonstrate slight variabilities among different ME films. Second, the reference voltage for the stimulation driver is generated on-chip; as a result, its accuracy may be affected by process variations in semiconductor fabrication. To ensure effective stimulation, we could calibrate the voltage reference generator with the downlink data to guarantee desired stimulating voltages in all cases.

### ME power transfer characterization

Ex vivo porcine tissue that consisted of pork chuck, purchased from a local store, was a general mixture of mostly muscle tissue mixed with some fat and connective tissue. The 15-turn magnetic coil was held in place vertically with a small ~3 mm air gap from the piece of tissue and the permanent bias magnet was placed ~2 cm away from the alternating current (AC) coil. To maintain the feedback pins on the device, the ME film was encapsulated in the endovascular 3D-printed capsule, implanted with a stiffener into the distal end of the ex vivo tissue and pushed through until the device reached the proximal end with the coil. The film was then used to power the ME-BIT, the various feedback signals were monitored and rectified voltage was recorded up to 4 cm deep within the tissue. To measure power transfer efficiency, we measured both the transmitter power as well as the peak implant power. Using a current probe (CT2 AC Current Probe, Tektronix) to measure current running through the AC coils, as well as a potentiostat (Gamry Reference 600+) to measure the impedance of the resonant coil and ferrite shield at the operating frequency (~0.5 Ω real impedance, where imaginary impedance was cancelled at the resonant frequency by choosing the corresponding capacitors for the inductance of the coil), the transmitter power was calculated (Supplementary Fig. 7). The peak implant power, on the other hand, was measured by observing the charge current on the device powered by an 8.75 mm^2^ ME film, where $I = \frac{{dV_{\mathrm{rect}}}}{{dt}} \times C$, where C (~800 pF) is the on-chip capacitor and $P_{\mathrm{in}} = V_{\mathrm{rect}} \times I$ (Supplementary Fig. 8). The peak implant power was held constant with the rectified voltage at 1.8 V, while the coil current was increased to sustain the operating voltage.

### Magnetic field transmitter

A microcontroller or computer that is capable of communicating via I2C protocol sends commands to a frequency synthesizer IC. The frequency synthesizer IC provides the three different data and notch frequencies used to communicate with the custom IC. This signal is then modulated through various circuits before being transmitted to half-bridge drivers that are then passed to an H-bridge made up of 4 integrated driver ICs and MOSFETS (CSD95378BQ5M, Texas Instruments). The H-bridge is rated up to 30 Amps continuous current with an upper frequency limit of 1.25 MHz. The data and notch frequencies, as well as all of the stimulation parameters, are set by the user through a serial interface in Arduino. The H-bridge is then connected to surface coils that are wrapped with 18 AWG litz wire (MWS Wire) and resonated with high-voltage rated capacitors (~6 kV, WIMA). We designed transmitter coils to provide uniform magnetic fields for characterization and a large alignment tolerance so that we could effectively power our devices in the operating room. On the basis of COMSOL simulations, we chose a spiral coil with an inner diameter of 6 cm, with 15 turns and an outer diameter of 7 cm. We chose this size because it would be compatible with a wearable transmitter system^38^. When the ME film was aligned parallel to the surface of the coil (as is the case for experiments in the operating room), we placed the coil off-centre from the ME-BIT to power the device with the fringing fields. The impedance of the coil was measured to be ~0.5 Ω. The field profiles were simulated using COMSOL and experimentally measured with an AC magnetic field probe (AMF Life Systems). The field profile was created by simulating a current of 18 A in the 15-turn coil. A more robust magnetic field driver was designed and assembled for use in the operating room for the porcine experiments. This driver used high-electron-mobility gallium-nitride transistors (GS61008T, GaN Systems) for the output H-bridge stage and had optimized magnetic board layout for high-power, high-frequency switching operation^60^. The COMSOL model for the angular misalignment analysis used a similarly sized 7-cm-diameter coil. A 5 mm × 1.75 mm × 0.023 mm Metglas sheet was placed within a 14 mm × 3 mm × 2.15 mm airbox to simulate the ME-BIT. To model how the device would behave in vivo, the ME-BIT was placed within a tissue layer model (20 mm of muscle, 5 mm of fat and 2 mm of skin) at the distance of 15 mm consistent with the large animal experiments. The device was then rotated in two different directions (*θ*, *φ*). For each angle, the transmitter was translated to the position that achieved peak voltage across the film. For example, at a 90° rotation, the ME-BIT was placed off-centre to maximize the use of the fringing fields. Because we operated at the linear region of the magnetostrictive curve, strain induced on the magnetostrictive layer is linearly related to the induced voltage on the ME film^38,61^. Thus, we used the simulated strain induced in the Metglas film to calculate the induced voltage and normalize this voltage to the peak value (Fig. 3d).

### In vivo rat stimulation model

All procedures complied with the National Institutes of Health standards and were approved by the Animal Care and Use Committee of Rice University (Protocol no. IACUC-20-181). In vivo stimulation with ME wireless power was confirmed in 3 different male Long-Evans rats (Charles River) weighing 300–400 g. The stimulation was verified with a visually observed leg kick and corresponding EMG recording in the subplantar region of the foot. For the acute procedure, the animal was placed in an induction chamber with 5% isoflurane in oxygen at a flow rate of 1–2 l min^−1^ until the rat was unconscious and areflexic, confirmed with toe pinches. The animal was then transferred to a 40 °C heated pad with a nose cone with ~2% isoflurane. Meloxicam (2 mg kg^−1^ subcutaneously) and Ethiqa XR (0.65 mg kg^−1^ subcutaneously) were administered to the rat before shaving the surgical site. Iodine swabs were used to sterilize the site before a single semi-circular incision was made across the lower hip of the rat. The fascial plane between the gluteus maximus and the anterior head of the bicep femoris was opened to expose the sciatic nerve. The underlying connective tissue was severed to better isolate the sciatic nerve.

Two EMG electrode needles were placed in the plantar muscles of the rat leg, while a third ground electrode was placed on the main body of the rat. The recording electrodes were connected to a dual bioamplifier (ADsystems) and sampled at 1 kHz. The data were acquired and exported through Labchart and processed in Matlab 2017. On completion of the study, the animals were immediately euthanized under proper guidelines.

### In vivo porcine model

The animal procedures were conducted in accordance with the rules of the IACUC (Protocol no. 2007074). Eight female Yorkshire pigs weighing approximately 35–45 kg received a 7 d acclimation period before any procedure. General anaesthesia was administered by veterinary services personnel and was established with Telazol (4.4 mg kg^−1^), ketamine (2.2 mg kg^−1^) and xylazine (2.2 mg kg^−1^ intramuscularly), followed by intubation under general anaesthesia. Mechanical ventilation was given with a mixture of oxygen and isoflurane (1–3%). Routine physiological monitoring was performed.

The pigs were placed in supine position and the femoral artery was palpated between the rectus femoris and the vastas medials muscles. A 6 cm skin incision was performed to expose the femoral neurovascular bundle and blunt dissection was used to remove the surrounding connective tissue and adventitia, further exposing the femoral artery, vein and nerve. Baseline EMGs (recorded from the quadricep muscles) and nerve action potentials (NAPs, recorded from the femoral nerve) were obtained from direct femoral nerve stimulation to ensure nerve integrity after exposure. Swine analogues to Human 10/20 electrode positions were placed: nasion (Ns), cervical spine rostral (CSr) and cervical spine caudal (CSd).

A 9 Fr sheath was then delivered into the common femoral artery through a modified Seldinger technique. A parylene insulated wire (0.008 in) connected to the ME implant was introduced into the vessel. NAPs were then recorded on the femoral nerve and EMGs on the adjacent quadricep muscles after endovascular femoral nerve stimulation. Leg twitching was observed with each EMG recording. Central signals were also recorded from the cranial and cervical electrodes. Next, under direct fluoroscopic visualization, a 5 Fr Mikelson catheter (Cook Medical) was advanced over a 0.035 in Glidewire (Terumo IS) into the descending aorta at the level of a segmental artery. The same parylene insulated wire connected to the ME implant was then introduced into the segmental artery through a microcatheter (0.017 in inner diameter). Endovascular DRG and intercostal nerve stimulation was then performed through wireless magnetoelectric stimulation of the microwire. EMG was recorded from the intercostal muscles and twitches were seen with each recording. On completion of the study, the animals were immediately euthanized under proper guidelines. The femoral and intercostal arteries tested were collected for histopathologic examination. All tissue samples were routinely fixed in 10% formalin, processed and embedded in paraffin. Tissue blocks were sectioned at 5 µm thickness for hematoxylin and eosin (H&E) and modified Movat pentachrome histochemical staining^62,63^.

The electrophysiologic recordings were done on a Cadwell IOMax utilizing subdermal needle electrodes for SSEP and EMG recordings. Direct nerve stimulation was performed via a triple hook electrode and direct nerve recordings were done via a double hook probe. Central response recordings were done via one subdermal needle placed over the rostrum, referenced to a subdermal needle placed over the midline of the cervical spine. In some cases, a second subdermal needle was placed over the cervical spine, with one positioned just behind the occiput and one placed at the lower cervical spine, always over the midline. A ground electrode was placed in the shoulder to help eliminate any unwanted artifacts. Amplification for central recordings and nerve action potentials was 100 µV per div, and 1,000 µV per div for all EMG recordings. Digital filter bandpass settings were applied for each type of recording: central sensor responses (30–500 Hz), EMG recordings (10–3,000 Hz) and nerve action potentials (30–1,000 Hz).

### Reporting Summary

Further information on research design is available in the Nature Research Reporting Summary linked to this article.

## Supplementary information

Supplementary Information Supplementary figures and table.

Reporting Summary

Peer Review Information

## Data Availability

The main data supporting the results in this study are available within the paper and its Supplementary Information. The raw data are available from the corresponding authors on reasonable request.

## Code availability

The Arduino teensy code used to drive magnetic fields can be found at https://github.com/RobinsonLab-Rice/EVNS.
