# Supplementary material for: A wireless millimetric magnetoelectric implant for the endovascular stimulation of peripheral nerves
Source: Nat Biomed Eng. 2022 Mar 31;6(6):706–16. doi: 10.1038/s41551-022-00873-7 (PMC9213237; doi:10.1038/s41551-022-00873-7)
Supplement: Supplementary file 1 — Supplementary figures and table. [file 41551_2022_873_MOESM1_ESM.pdf]

nature portfolio

https://doi.org/10.1038/s41551-022-00873-7

Supplementary information

A wireless millimetric magnetoelectric implant for the endovascular stimulation of peripheral nerves

In the format provided by the authors and unedited

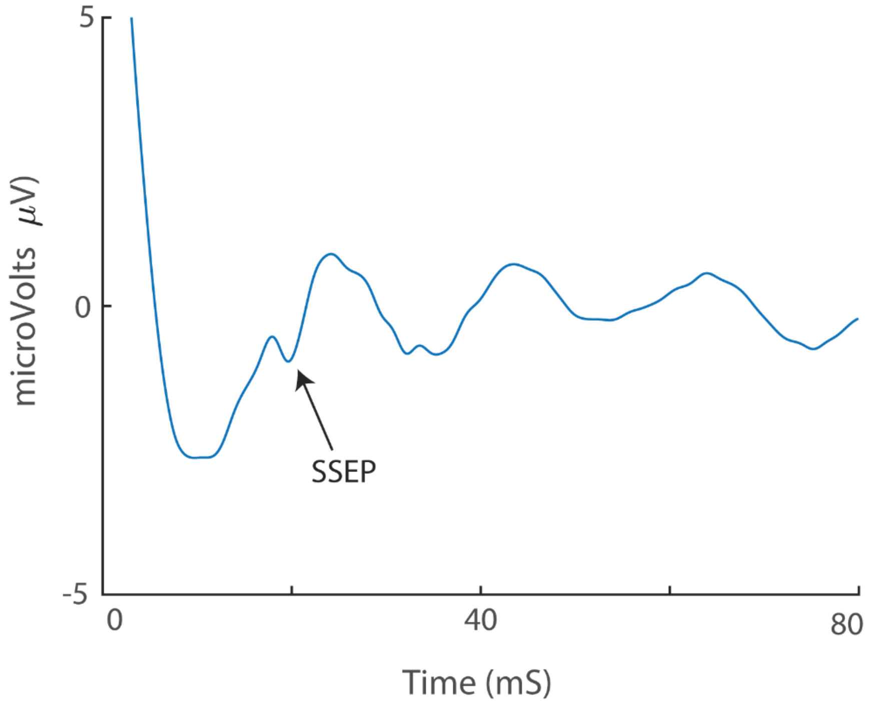

Supplementary Fig. 1 l SSEP Central recording of the brain stem in response to femoral nerve stimulation

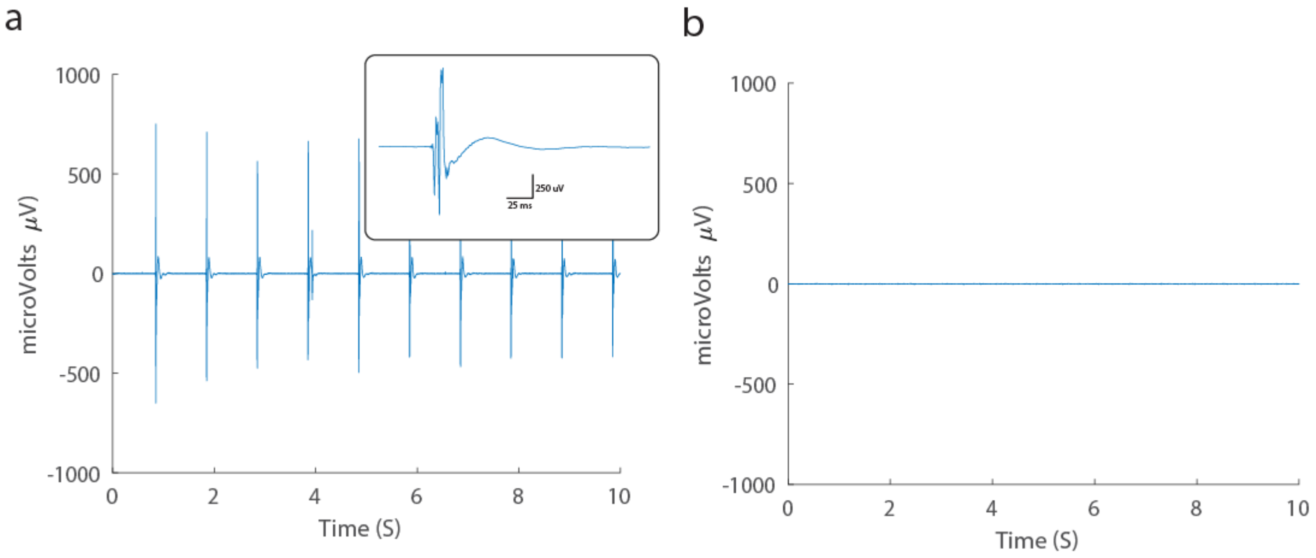

Supplementary Fig. 2 l (a) EMG recording for intercostal/DRG nerve stimulation. **(b)** EMG recording of control by using off-resonant magnetic field.

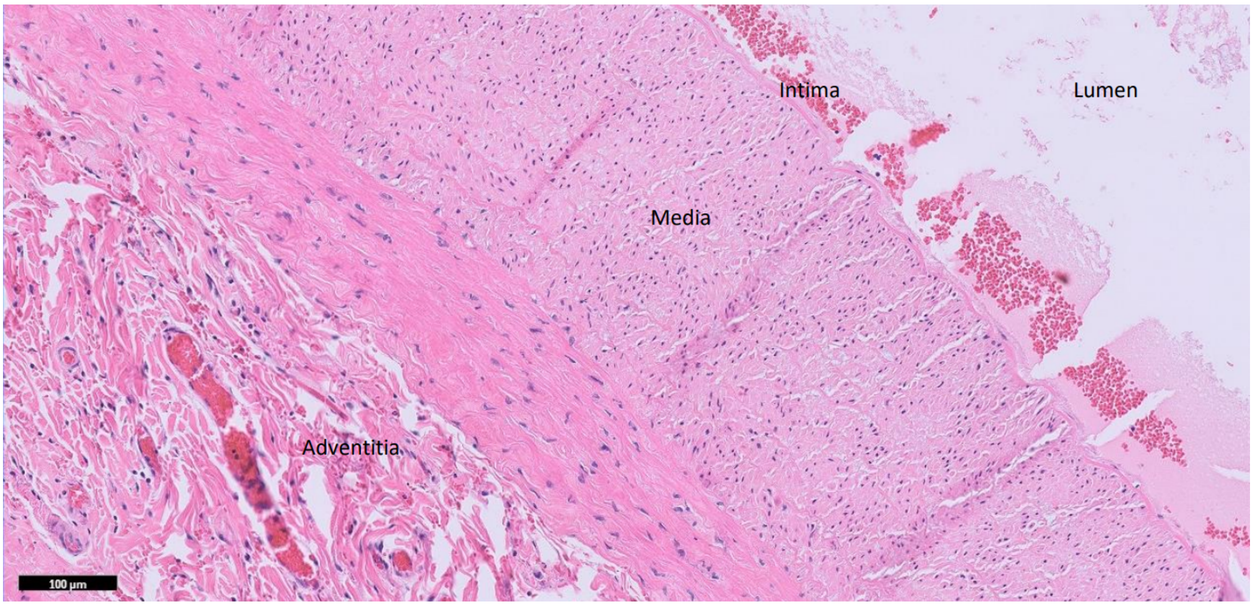

Supplementary Fig. 3 l H&E stain of femoral artery which shows no evidence of acute vascular damage.

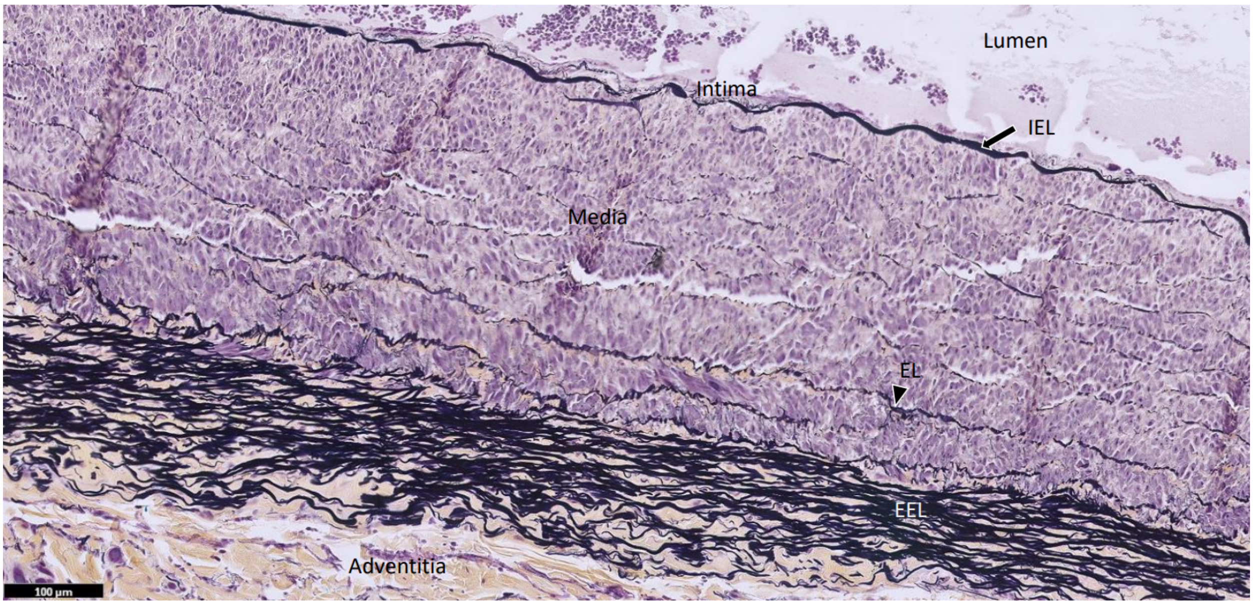

Supplementary Fig. 4 l Modified Movat stain of femoral artery which also shows no evidence of acute vascular damage; Movat stain highlights normal connective and elastic tissue of the artery. (IEL = internal elastic lamina; EL = elastic lamellae; EEL= external elastic lamina)

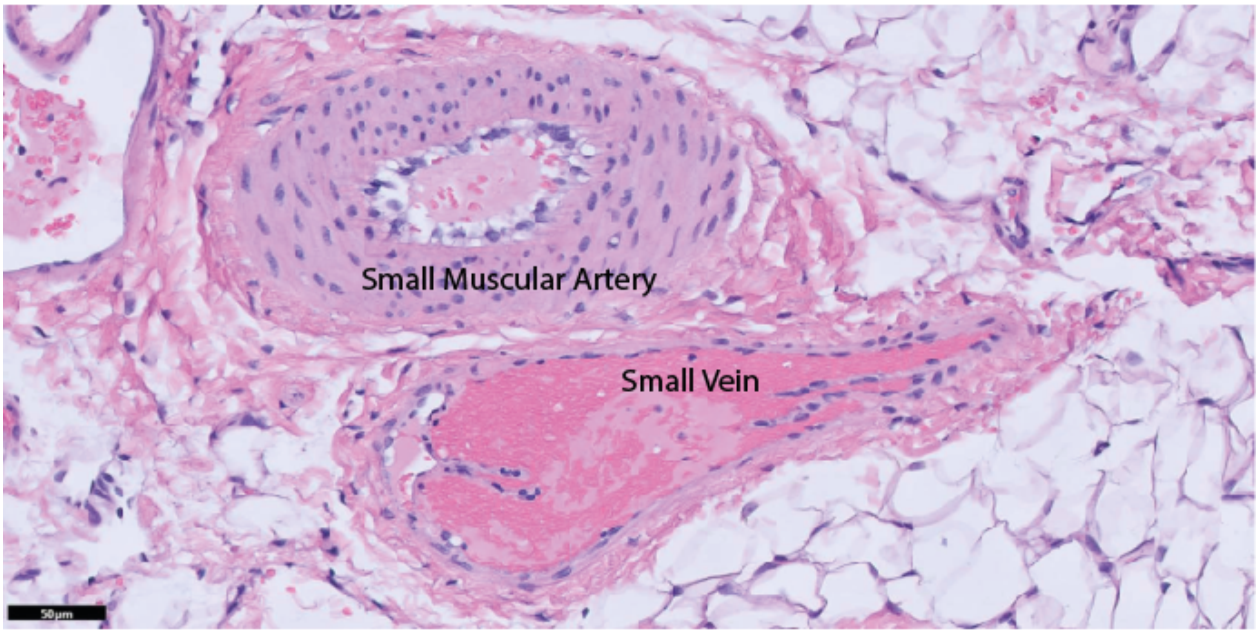

Supplementary Fig. 5 I H&E stain showing normal histology of the small vascular branches of an intercostal neurovascular bundle.

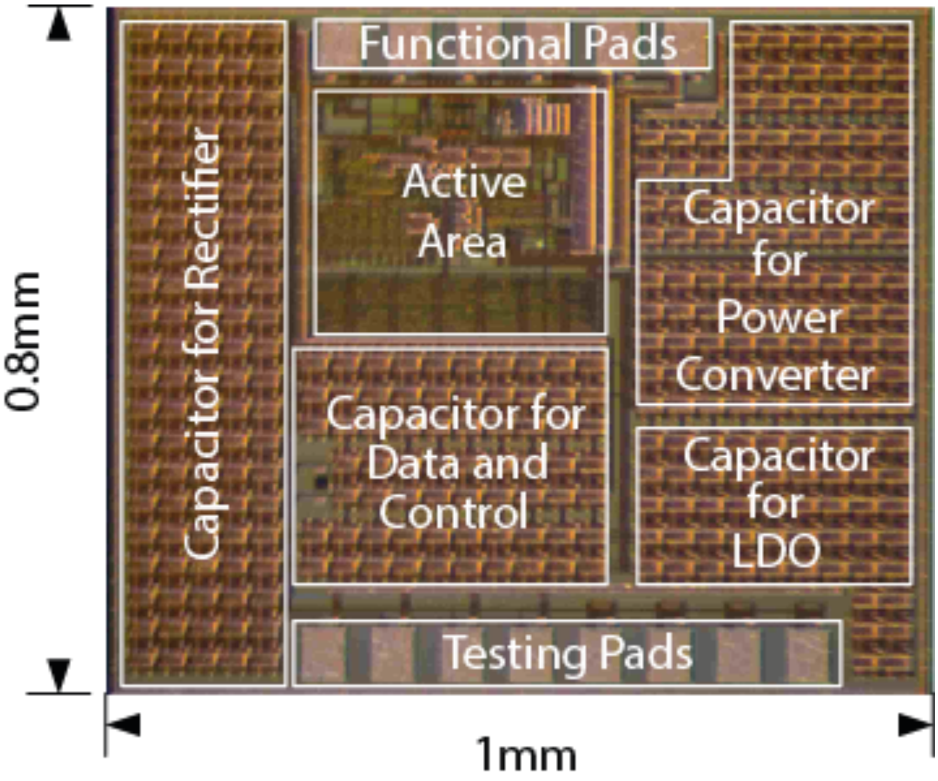

Supplementary Fig. 6 I Custom ASIC footprint and layout.

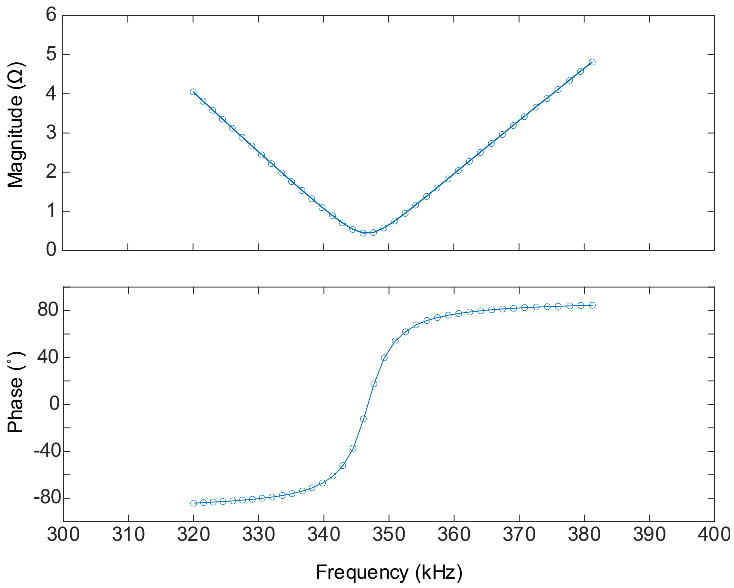

Supplementary Fig. 7 l Impedance magnitude and phase of the resonant surface coil used to characterize power transfer efficiency. Resonant frequency of the transmitting coil is around 345 kHz

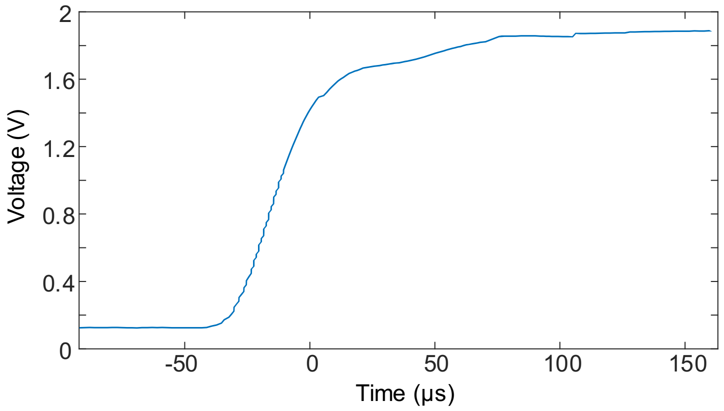

Supplementary Fig. 8 l Charging curve to the minimum operating voltage of ~1.8 V with measured rectified voltage of the ME-BIT.

TX-RX Distance (mm)	Coil Current (A)	Peak Implant Power (mW)	Power Transfer Efficiency (%)
0	0.23	1.17	4.4
5	0.34	1.17	1.98
10	0.65	1.17	0.55
20	1.4	1.17	0.12
30	3.63	1.17	0.01
40	8.6	1.17	0.003

Table 1 | Power transfer efficiency measurements in ex-vivo tissue. The peak implant power was held constant with the measured rectified voltage at 1.9 V, while the coil current was increased to sustain the operating voltage up to 40 mm.
