# Supplementary material for: A wireless millimetric magnetoelectric implant for the endovascular stimulation of peripheral nerves
Source: Nat Biomed Eng. 2022 Mar 31;6(6):706–16. doi: 10.1038/s41551-022-00873-7 (PMC9213237; doi:10.1038/s41551-022-00873-7)
Supplement: Supplementary file 2 — Reporting Summary [file 41551_2022_873_MOESM2_ESM.pdf]

nature portfolio

Corresponding author(s): Jacob T. Robinson, Kaiyuan Yang and Sunil A. Sheth

Last updated by author(s): Jan 16, 2022

Reporting Summary

Nature Portfolio wishes to improve the reproducibility of the work that we publish. This form provides structure for consistency and transparency in reporting. For further information on Nature Portfolio policies, see our Editorial Policies and the Editorial Policy Checklist.

Statistics

For all statistical analyses, confirm that the following items are present in the figure legend, table legend, main text, or Methods section.

n/a	Confirmed	
☐	☒	The exact sample size (*n*) for each experimental group/condition, given as a discrete number and unit of measurement
☐	☒	A statement on whether measurements were taken from distinct samples or whether the same sample was measured repeatedly
☒	☐	The statistical test(s) used AND whether they are one- or two-sided<br>*Only common tests should be described solely by name; describe more complex techniques in the Methods section.*
☒	☐	A description of all covariates tested
☐	☒	A description of any assumptions or corrections, such as tests of normality and adjustment for multiple comparisons
☐	☒	A full description of the statistical parameters including central tendency (e.g. means) or other basic estimates (e.g. regression coefficient) AND variation (e.g. standard deviation) or associated estimates of uncertainty (e.g. confidence intervals)
☒	☐	For null hypothesis testing, the test statistic (e.g. *F*, *t*, *r*) with confidence intervals, effect sizes, degrees of freedom and *P* value noted<br>*Give P values as exact values whenever suitable.*
☒	☐	For Bayesian analysis, information on the choice of priors and Markov chain Monte Carlo settings
☒	☐	For hierarchical and complex designs, identification of the appropriate level for tests and full reporting of outcomes
☒	☐	Estimates of effect sizes (e.g. Cohen's *d*, Pearson's *r*), indicating how they were calculated

Our web collection on statistics for biologists contains articles on many of the points above.

Software and code

Policy information about availability of computer code

Data collection	Arduino code was used to program the microcontroller and signal generators that were used to control the magnetic-field drivers. The experimental data in rats were collected with a dual amplifier DAQ system from AD systems. The raw data were visualized through LabChart and post-processed in Matlab R2021a. The porcine data were taken with a Cadwell IOMax recording system and visualized through its built-in software, and then post-processed in Matlab R2021a and Matlab R2017a. The arduino teensy code used to drive magnetic fields can be found at https://github.com/RobinsonLab-Rice/EVNS.
Data analysis	Data were analysed via custom scripts in Matlab R2021a and Matlab R2017a.

For manuscripts utilizing custom algorithms or software that are central to the research but not yet described in published literature, software must be made available to editors and reviewers. We strongly encourage code deposition in a community repository (e.g. GitHub). See the Nature Portfolio guidelines for submitting code & software for further information.

Data

Policy information about availability of data

All manuscripts must include a data availability statement. This statement should provide the following information, where applicable:

- Accession codes, unique identifiers, or web links for publicly available datasets
- A description of any restrictions on data availability
- For clinical datasets or third party data, please ensure that the statement adheres to our policy

The main data supporting the results in this study are available within the paper and its Supplementary Information. The raw data are available from the corresponding authors on reasonable request.

March 2021

Field-specific reporting

Please select the one below that is the best fit for your research. If you are not sure, read the appropriate sections before making your selection.

☒ Life sciences ☐ Behavioural & social sciences ☐ Ecological, evolutionary & environmental sciences

For a reference copy of the document with all sections, see nature.com/documents/nr-reporting-summary-flat.pdf

Life sciences study design

All studies must disclose on these points even when the disclosure is negative.

Sample size	For the rat experiments, two different rats were used, and stimulation was confirmed in both animals by the ME-BIT via visual observation of leg kicks as well as measured EMG signals. For the large-animal experiments, data are shown for one pig; additional animals were used in pilot studies. This study was designed to show the capabilities of the ME-BIT; clear physiological responses were observed and the relevant controls were run to demonstrate efficacy.
Data exclusions	No data were excluded from the analyses.
Replication	Specific experimental findings in the rat model were reproduced across different animals. For the large animal model, stimulation was reproduced with one pig (that is, the stimulation of the femoral nerve and the segmental nerve were readily replicated within the same animal).
Randomization	Randomization was not relevant for the study, as successful stimulation and EMG showed a physiological response to the controlled endovascular stimulation.
Blinding	Blinding was not used in this study.

Reporting for specific materials, systems and methods

We require information from authors about some types of materials, experimental systems and methods used in many studies. Here, indicate whether each material, system or method listed is relevant to your study. If you are not sure if a list item applies to your research, read the appropriate section before selecting a response.

Materials & experimental systems

n/a	Involved in the study	
☒	☐	Antibodies
☒	☐	Eukaryotic cell lines
☒	☐	Palaeontology and archaeology
☐	☒	Animals and other organisms
☒	☐	Human research participants
☒	☐	Clinical data
☒	☐	Dual use research of concern

Methods

n/a	Involved in the study	
☒	☐	ChIP-seq
☒	☐	Flow cytometry
☒	☐	MRI-based neuroimaging

Animals and other organisms

Policy information about studies involving animals; ARRIVE guidelines recommended for reporting animal research

Laboratory animals	Adult male Long Evans rats (300–400g) were sourced from Charles Rivers.<br>The female Yorkshire pigs weighed 35–45kg.
Wild animals	The study did not involve wild animals.
Field-collected samples	The study did not involve samples collected from the field.
Ethics oversight	All in vivo experiments were performed in accordance with the rules of the IACUC at Rice University and at the University of Texas Medical Branch at Galveston.

Note that full information on the approval of the study protocol must also be provided in the manuscript.
