# Supplementary material for: A wireless millimetric magnetoelectric implant for the endovascular stimulation of peripheral nerves
Source: Nat Biomed Eng. 2022 Mar 31;6(6):706–16. doi: 10.1038/s41551-022-00873-7 (PMC9213237; doi:10.1038/s41551-022-00873-7)
Supplement: Supplementary file 3 — Peer Review Information [file 41551_2022_873_MOESM3_ESM.pdf]

nature biomedical engineering | PEER-REVIEW INFORMATION

A wireless millimetric magnetoelectric implant for the endovascular stimulation of peripheral nerves

Corresponding author: Jacob Robinson

Editorial note

This document includes relevant written communications between the manuscript's corresponding author and the editor and reviewers of the manuscript during peer review. It includes decision letters relaying any editorial points and peer-review reports, and the authors' replies to these (under 'Rebuttal' headings). The editorial decisions are signed by the manuscript's handling editor, yet the editorial team and ultimately the journal's Chief Editor share responsibility for all decisions.

Any relevant documents attached to the decision letters are referred to as **Appendix #**, and can be found appended to this document. Any information deemed confidential has been redacted or removed. Earlier versions of the manuscript are not published, yet the originally submitted version may be available as a preprint. Because of editorial edits and changes during peer review, the published title of the paper and the title mentioned in below correspondence may differ.

Correspondence

Mon 02 Aug 2021
Decision on Article nBME-21-1511

Dear Dr Robinson,

Thank you again for submitting to *Nature Biomedical Engineering* your manuscript, "Wireless endovascular nerve stimulation with a millimeter-sized magnetoelectric implant". The manuscript has been seen by three experts, whose reports you will find at the end of this message. You will see that the reviewers appreciate the work, and that Reviewers #1 and #3 raise a few technical points that we hope you will be able to address. In particular, we would expect that a revised version of the manuscript provides:

* Quantitative evidence of in vivo performance under angular misalignment with the transmitter.

* Discussion of the miniaturization and translatability challenges for the device.

* Additional device characterization, as per the comments of Reviewers #1 and #3.

* Thorough methodological descriptions, as per the comments of Reviewers #1 and #3.

When you are ready to resubmit your manuscript, please upload the revised files, a point-by-point rebuttal to the comments from all reviewers, the reporting summary, and a cover letter that explains the main improvements included in the revision and responds to any points highlighted in this decision.

Please follow the following recommendations:

* Clearly highlight any amendments to the text and figures to help the reviewers and editors find and understand the changes (yet keep in mind that excessive marking can hinder readability).

* If you and your co-authors disagree with a criticism, provide the arguments to the reviewer (optionally,

Open Access This file is licensed under a Creative Commons Attribution 4.0 International License, which permits use, sharing, adaptation, distribution and reproduction in any medium or format, as long as you give appropriate credit to the original author(s) and the source, provide a link to the Creative Commons license, and indicate if changes were made. In the cases where the authors are anonymous, such as is the case for the reports of anonymous peer reviewers, author attribution should be to 'Anonymous Referee' followed by a clear attribution to the source work. The images or other third-party material in this file are included in the article's Creative Commons license, unless indicated otherwise in a credit line to the material. If material is not included in the article's Creative Commons license and your intended use is not permitted by statutory regulation or exceeds the permitted use, you will need to obtain permission directly from the copyright holder. To view a copy of this license, visit http://creativecommons.org/licenses/by/4.0.

indicate the relevant points in the cover letter).

* If a criticism or suggestion is not addressed, please indicate so in the rebuttal to the reviewer comments and explain the reason(s).

* Consider including responses to any criticisms raised by more than one reviewer at the beginning of the rebuttal, in a section addressed to all reviewers.

* The rebuttal should include the reviewer comments in point-by-point format (please note that we provide all reviewers will the reports as they appear at the end of this message).

* Provide the rebuttal to the reviewer comments and the cover letter as separate files.

We hope that you will be able to resubmit the manuscript within 15 weeks from the receipt of this message. If this is the case, you will be protected against potential scooping. Otherwise, we will be happy to consider a revised manuscript as long as the significance of the work is not compromised by work published elsewhere or accepted for publication at *Nature Biomedical Engineering*.

We hope that you will find the referee reports helpful when revising the work, which we look forward to receive. Please do not hesitate to contact me should you have any questions.

Best wishes,

Pep

—
Pep Pàmies
Chief Editor, *Nature Biomedical Engineering*

____
Reviewer #1 (Report for the authors (Required)):

The manuscript is clear and well written, and the technology well characterized. However, the proposed research suffers from 2 major weaknesses:

1) A similar device has been presented in prior publications by the same lab (e.g., Amanda et al, Cell, 2020). It seems that the presented technology only shows minor differences. If that is not the case then the authors should place more effort in stating what is new. If the novelty mainly comes from the change in application then the authors should focus on the following 2nd major weakness.

2) The proof-of-concept does not show the capability of the device to stimulate within blood vessels. The targeted application requires very small implants. However, the presented implantable device is currently too bulky to fit within blood vessels. If this is not the case, then why was this not demonstrated in vivo? Miniaturization is not trivial, and I'm concerned that most characterization plots will no longer apply once the ME antenna is made smaller. For instance, Fig 3(e,f) will be very different, and powering at large distances (>3cm) might no longer be possible. State-of-the-art ultra-small stimulating implants are much smaller (sub-mm) than the proposed technology that is currently in the mm scale.

Specific comments that the authors should address include:

• Line 30: This is the case for commercial devices but what about technologies developed in academic labs? The authors should mention the state-of-the-art sub-cm sized devices and novel minimally invasive surgical techniques (e.g., Cortese et al 2020, Khalifa et al 2021), mention their weaknesses, and explain the benefits of using the proposed technology. The reader might wonder: if the implant is very small and can be fully injected, then why use the vascular route which could lead to complications?

• Line 53 and 58: This depends on the size of the implant.

• Line 78: Not entirely sure this is true, please check the work by John Ho et al.

• Line 90: Please add a reference.

• Line 136: How was the surface coil designed? What specs were targeted?

• Line 173 and 233: Please provide information on transmitted power and powering distance.

• Line 246: Since the device is an implant, measured PTE should be done with tissue.

• Line 256: Since the ME antenna is the main novelty it would be interesting to discuss how it compares to other miniaturized implants that utilize ME antennas.

• Line 296: Details about the electrode should be provided.

• Line 392: The strength of the research lies in this experiment. However, since the implant is bulky the surgery is not minimally invasive which makes the concept less appealing.

• Line 411: This is the biggest weakness of the research. Although the ME antenna shows multiple benefits over other types of powering methods it does not mean the device has "excellent scaling properties". There are multiple challenges of scaling an implant, for instance, new packaging techniques will need to be applied, the operating distance will be significantly reduced, the techniques used to make the ME antenna will change as it might have to be microfabricated, the connection between the IC and the electrode will be more challenging, etc. The authors should address some of these challenges instead of simply showing the very high PTE of a relatively large ME antenna.

Reviewer #2 (Report for the authors (Required)):

* A brief summary of the results:
Magnetoelectrics hold promise to enable miniaturization of electronics for blood vessels.
Fabricated an ASIC encapsulated in a 3 x 2.15 x 14.8 mm package.
A custom magnetic field transmitter was used – at up to 4cm depths.
Can generate up to 4 mW of >8 Vpp.
ASIC that uses the digitally received data to program the shape (mono-phasic or bi-phasic), the amplitude (0.3 V to 3.3 V with 4-bit resolution), the pulse width (0.05 ms to 1.2 ms with 3-bit resolution), and the delay (0.01 ms to 0.8 ms) of the stimulation.
The ASIC, fabricated on 180 nm complementary metal-oxide-semiconductor (CMOS) technology (TSMC), measures only 1 by 0.8 mm.
ME-BITs can tolerate approximately 3 cm misalignment from the center of the transmitter coil and a depth of 3 cm in tissue.
Deployed our ME-BIT through an 9Fr sheath into the femoral artery.
ME-BIT can be implanted deep within the tissue close to targeted areas without requiring lead wires that connect to a more superficial inductive coil.
First example of a magnetoelectric-powered bioelectronic implant in a large animal model.

* Your reasoned opinion on the degree of advance (fundamental, mechanistic, methodological, technological, therapeutic, translational and/or clinical) of the work with respect to the state of the art. If the results or conclusions are not original, please provide relevant references:

This work represents a major technological advancement that solves a clinical problem. More work needs to be done to answer questions regarding viability for clinical delivery but the foundational science and validation data are extremely encouraging.

* Your reasoned opinion on the broad implications of the findings:

Solving the problems of wireless power delivery to enable minimally invasive / endovascular stimulation could create a new industry. This solution, although early, shows potential as a significant breakthrough.

* Any major technical criticisms or questions.
none

* Any minor technical criticisms or questions.
None

Reviewer #3 (Report for the authors (Required)):

The paper reports on a mm-scale neural stimulation implant that is powered wirelessly. The wireless power receiver is a magnetoelectric laminate (Metglas-PZT). The implant's size is such that it could feasibly, with more engineering, be implanted inside a blood vessel and be delivered endovascularly. The implant was demonstrated on both small and large animal models.

The manuscript is well-written and well-organized. The system is technologically impressive and the animal model demonstrations are compelling. 4 mW of power at a receiver of size 0.3 X 1.75 X 5 mm is very impressive and compares well with the state of the art. This is a high quality manuscript that I believe will be of interest to readers and demonstrates significant technological advancements to the state of the art.

I have a few minor questions and recommendations as described below.

1. As PZT has lead, it might be worthwhile to address the safety of PZT implants.

2. 1 mT at ~ 345 kHz is a large field and exceeds both IEEE and ICNIRP safety standards. (These standards have been recently updated safe fields at this frequency have been increased, but not up to 1 mT.) The authors should at least address the safety issues of transmitting fields > 1 mT at these frequencies into the human body.

3. What specific Metglas formulation is used? Is the Metglas prepared in any way (i.e. annealed, pre-magnetized)?

4. The vibration mode of the ME device is not clear. Given the size and frequency, I assume an extensional vibration mode. There would be other modes, such as a primary bending mode at different frequencies. Did the authors do a full frequency sweep of the response of the ME device? Figure 2 only shows 250 – 450 kHz. Why choose this frequency range?

5. How is the device, and specifically the ME film, encapsulated for the experiment in the large animal model? It looks like the ME film is directly encapsulated in the epoxy without any air gap. If this is the case, I suspect that the epoxy is significantly dampening the vibrations of the film. Better performance might be obtained by encapsulating the ME film so that it can vibrate more freely.

6. The text on the figures (e.g. Fig. 2) is very small and hard to read.

7. On line 207, the definition of stimulation efficiency (n_stim = stimulation amplitude / stimulation supply) is a little odd to me. Perhaps this is a standard definition for implant stimulation, but efficiency is typically defined with power or energy ratios, not voltage ratios. A high stimulation efficiency, defined as voltage ratios, does not necessarily imply that heating won't be an issue.

8. The authors demonstrate via simulation and experimentation that the implant is robust to 3 cm of lateral misalignment. That is good. However, their arguments about angular misalignment are weaker. They simply claim better angular misalignment than other mm-scale technologies by referring to another study. I would suggest that the authors either leave out the paragraph about angular misalignment (lines 253 – 259), which is not really critical to the paper anyway, or provide quantitative simulation based angular misalignment performance.

9. Line 419. I'm not so sure that magnetic fields at 350 kHz will easily penetrate metal casings. Glass and ceramic should be fine.

Wed 12 Jan 2022
Decision on Article nBME-21-1511

Dear Dr Robinson,

Thank you for your patience in waiting for our feedback on your revised manuscript, "Wireless endovascular nerve stimulation with a millimeter-sized magnetoelectric implant". Reviewer #1 has unfortunately not delivered a report, despite our multiple chasers, and Reviewer #2 declined to re-review. Nevertheless, on the basis of our judgment of all the reports received for this manuscript and your responses to the criticisms, and having consulted with Reviewer #3, who has no further concerns, I am pleased to write that we shall be happy to publish the manuscript in *Nature Biomedical Engineering*, provided that the points specified in the attached instructions file are addressed.

When you are ready to submit the final version of your manuscript, please upload the files specified in the instructions file.

For primary research originally submitted after December 1, 2019, we encourage authors to take up transparent peer review. If you are eligible and opt in to transparent peer review, we will publish, as a single supplementary file, all the reviewer comments for all the versions of the manuscript, your rebuttal letters, and the editorial decision letters. **If you opt in to transparent peer review, in the attached file please tick the box 'I wish to participate in transparent peer review'; if you prefer not to, please tick 'I do NOT wish to participate in transparent peer review'.** In the interest of confidentiality, we allow redactions to the rebuttal letters and to the reviewer comments. If you are concerned about the release of confidential data, please indicate what specific information you would like to have removed; we cannot incorporate redactions for any other reasons.
More information on transparent peer review is available.

Please do not hesitate to contact me should you have any questions.

Best wishes,

Pep

—
Pep Pàmies
Chief Editor, *Nature Biomedical Engineering*

Reviewer #3 (Report for the authors (Required)):

Thank you for your detailed response to my recommendations, all of which have been addressed. I have no further comments or recommendations.

Nature Biomedical Engineering is a Transformative Journal. Authors may publish their research with us through the traditional subscription access route, or make their paper immediately open access through payment of an article-processing charge. More information about publication options is available.

You may need to take specific actions to comply with funder and institutional open-access mandates. If the work described in the accepted manuscript is supported by a funder that requires immediate open access (as outlined, for example, by Plan S) and your manuscript was originally submitted on or after January 1st 2021, then you will need to select the gold OA route. Authors selecting subscription publication will need to

accept our standard licensing terms (including our self-archiving policies), and these will supersede any other terms that the author or any third party may assert apply to any version of the manuscript.

Rebuttal 1

Editors Comments:

Thank you again for submitting to Nature Biomedical Engineering your manuscript, “Wireless endovascular nerve stimulation with a millimeter-sized magnetoelectric implant”. The manuscript has been seen by three experts, whose reports you will find at the end of this message. You will see that the reviewers appreciate the work, and that Reviewers #1 and #3 raise a few technical points that we hope you will be able to address. In particular, we would expect that a revised version of the manuscript provides:

* Quantitative evidence of in vivo performance under angular misalignment with the transmitter.

* Discussion of the miniaturization and translatability challenges for the device.

* Additional device characterization, as per the comments of Reviewers #1 and #3.

* Thorough methodological descriptions, as per the comments of Reviewers #1 and #3.

Response to editor: We thank Dr. Pàmies for his efforts in gathering these reviews and the opportunity to improve this manuscript by addressing them. We also greatly appreciate all the insightful comments and feedback from the reviewers. Below, we have summarized the major changes, provided a list of new experiments,simulations and analysis, and included a point-by-point response to the reviewers with revisions to the manuscript italicized in the responses and revisions in the manuscript highlighted in red:

Summary of Major Changes:

1. **Added quantitative data for how angular misalignment affects *in-vivo* performance:**

   In order to determine the effects of angular misalignment in an in-vivo setting, we included a COMSOL simulation of the ME-BIT implanted within tissue as suggested by Reviewer 3. The updated data was included into the main **figure 3d**.

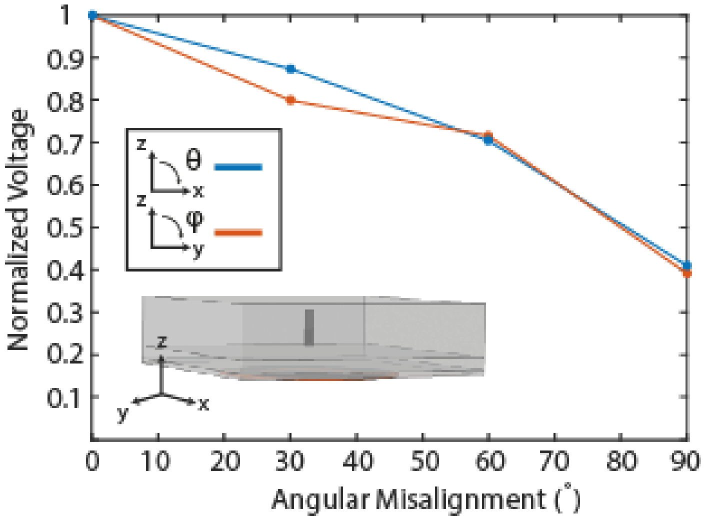

Fig. 3d. COMSOL simulation of the ME-BIT placed within a layered block of tissue (20 mm muscle, 5 mm fat, 2 mm skin) is used to model angular misalignment tolerances in-vivo for both θ and φ angular rotations.

The main text was also updated to include a discussion of the results in line 285:

"To assess the angular tolerance of the ME-BIT in vivo, we used a COMSOL model to simulate how ME voltage is affected when it undergoes angular misalignment in tissue (see methods). Because the simulated coil is radially symmetrical, we found that rotating the film in either the θ direction, as shown in Fig. 3d, or in the φ angular direction, resulted in the ME voltage decaying similarly with either angular change and being able to maintain > 40% of the maximum voltage at a 90° rotation."

We also added the methodology for this experiment in line 589:

"The COMSOL model for the angular misalignment analysis used a similarly sized 7 cm diameter coil. A 5 mm x 1.75 mm x 0.023 mm Metglas sheet is placed within a 14 mm x 3 mm x 2.15 mm airbox to simulate the ME-BIT. To model how the device would behave in vivo, the ME-BIT is placed within a tissue layer model (20 mm of muscle, 5 mm of fat, and 2 mm of skin) at the distance of 15 mm consistent with the large animal experiments. The device is then rotated in two different directions (θ, φ). For each angle, the transmitter is translated to the position that achieves peak voltage across the film. For example, at a 90° rotation, the ME-BIT is placed off-center to maximize the use of the fringing fields. Because we operate at the linear region of the magnetostrictive curve, strain induced on the magnetostrictive layer is linearly related to the induced voltage on the ME film. [38, 59] Thus, we use the simulated strain induced in the Metglas film to calculate the induced voltage and normalize this voltage to the peak value. (Fig. 3d)"

2. **Added further discussion regarding challenges for miniaturization and translation.**

We added further discussion related to miniaturization and translation of the device, specifically covering topics brought up by reviewers #1 and #3. We included new text that discussed potential issues with translation such as safety concerns with materials and safety limits for electric fields and SAR within the human body. Future miniaturization challenges are also discussed including how ME films scale and what we expect the tradeoffs will be. New text includes:

In line 455: "*Future studies are needed to determine how chronic deployment of the ME-BIT within the blood vessel could affect vasculature health as well as the biocompatibility of the device, including if a hermetically sealed capsule is suitable for long term implantation of the lead-containing PZT or if there are suitable piezoelectric alternatives, such as PVDF that do not contain lead [37]. Another safety concern for the long-term implantation of the ME-BIT is the interactions between the applied magnetic field and biological tissue. Our COMSOL simulations show that a field of 1 mT at an implant depth of 3 cm corresponds to a surface magnetic field of 7.7 mT, which results in an electric field and SAR that are within the IEEE safety limit of 101 V/m and 2 W/kg for unrestricted environments [36]. For other guidelines such as by ICNIRP that have lower limits for*

magnetic field exposure, this device operates outside the compliance range [54]. Thus, future approval for these devices may depend on which standards are applied by the regulatory body. While we operate our device at an optimal rectified voltage of 2.5 V, the ME-BIT remains operational at voltages as low as 1.8 V, in which field strengths as low as 0.6–0.8 mT can still be used. Additionally, improvements to the ME materials that increase the PTE or reduced power consumption by the ASIC could allow these devices to operate with lower magnetic field strengths, which could make the devices compliant with additional safety standards."

In line 472: "*As we miniaturize the implant and ME film sizes, we expect that the ME-BITs will be able to still function at centimeter depths in tissue. This is because the ME film voltage does not depend on the area of the film [37,43]. As a result, we expect that received power will only decrease linearly with the size of the film. The film voltage, on the other hand, is expected to remain constant, which will ensure that the voltages are large enough to operate the ASIC. Thus, we expect the major effect of miniaturization would be longer charging times between stimulation pulses, which could decrease the maximum stimulation bandwidth. Future work must also address packaging and connectorization, which will likely need to be changed as devices approach sub-mm length scales. These efforts will be needed to compare ME-powered implants with other types of sub-mm-sized battery-free implants and their compatibility with new minimally invasive delivery techniques, which are promising bioelectronic technologies but have yet to demonstrate neural stimulation in a large animal model [53-55]."*

3. Updated experimental data for power transfer efficiency done in an ex-vivo model:

We added experimental data characterizing the power transfer efficiency of the ME-BIT within tissue. We also clarified the method for taking these power transfer efficiency measurements along with further characterization of the system including impedance of the transmitter coil along with the charging power of the ME-BIT. The new experimental data for the power transfer efficiency is presented and updated in the main figure 3. Other transmitter characterization data such as the impedance graph used to calculate the transmitter power along with the rectified voltage charging curve of the implant, used to calculate the peak implant power, is included as supplemental figures.

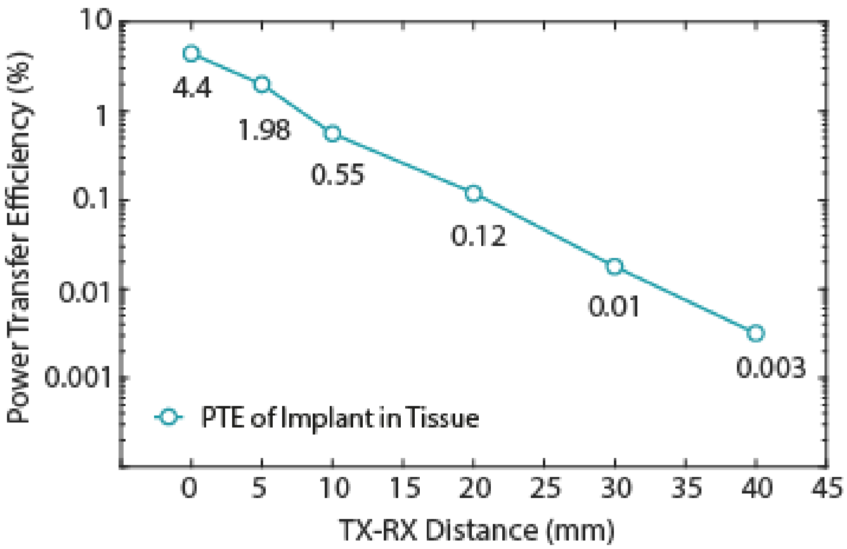

Fig. 3f. Measured power transfer efficiency for the magnetoelectric implant as a function of distance in tissue. At an operating distance of 30 mm, the transmitter power was ~ 6W to maintain the 1.17 mW implant power yielding a 0.01% efficiency

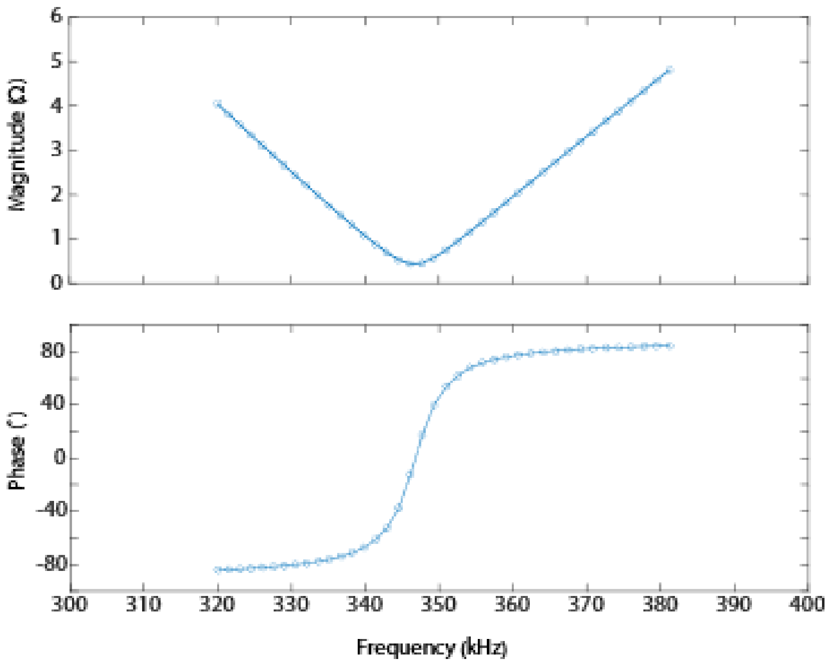

Supplemental Fig. 7 Impedance magnitude and phase of the resonant surface coil used to characterize power transfer efficiency. Resonant frequency of the transmitting coil is around 345 kHz

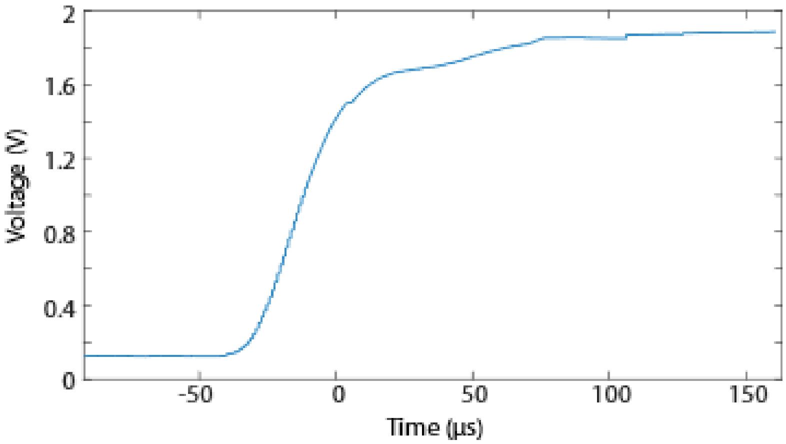

Supplemental Fig. 8 Charging curve to the minimum operating voltage of ~1.8V with measured rectified voltage of the ME-BIT.

TX-RX Distance (mm)	Coil Current (A)	Peak Implant Power (mW)	Power Transfer Efficiency (%)
0	0.23	1.17	4.4
5	0.34	1.17	1.98
10	0.65	1.17	0.55
20	1.4	1.17	0.12
30	3.63	1.17	0.01
40	8.6	1.17	0.003

Table 1. Power transfer efficiency measurements in ex-vivo tissue. The peak implant power was held constant with the measured rectified voltage at 1.9V, while the coil current was increased to sustain the operating voltage up to 40 mm.

4. Updated methods section with more in depth methodology:

We included more details on the methodology for several parts of our experiments including ME-BIT fabrication, measurements for power transfer efficiency, and design parameters used for our magnetic coils. The major text changes are included below:

In line 521: *"The assembled device is then placed within a 3D printed air-filled PLA capsule which allows the film to freely vibrate in air. The entire capsule is then sealed with non-conductive epoxy to provide more structural stability and prevent moisture from infiltrating the device. The assembled implant's final dimensions are 3 x 2.15 x 14.8 mm."*

In line 576: *"We designed transmitter coils to provide uniform magnetic fields for characterization and provide a large alignment tolerance so that we could effectively power our devices in the operating room (OR). Based on COMSOL simulations we chose a spiral coil with an inner diameter of 6 cm with 15 turns and an outer diameter of 7 cm. We chose this size because it would be compatible with a wearable transmitter system [38], and when the ME film is aligned parallel to the surface of the coil (as is the case for experiments in the OR) we place the coil off-center from the ME-BIT to power the device with the fringing fields. The impedance of the coil was measured to be ~0.5 Ω."*

Point-by-point responses:

Reviewer #1 (Report for the authors (Required)):

The manuscript is clear and well written, and the technology well characterized. However, the proposed research suffers from 2 major weaknesses:

1) A similar device has been presented in prior publications by the same lab (e.g., Amanda et al, Cell, 2020). It seems that the presented technology only shows minor differences. If that is not the case then the authors should place more effort in stating what is new. If the novelty mainly comes from the change in application then the authors should focus on the following 2nd major weakness.

2) The proof-of-concept does not show the capability of the device to stimulate within blood vessels. The targeted application requires very small implants. However, the presented implantable device is currently too bulky to fit within blood vessels. If this is not the case, then why was this not demonstrated in vivo? Miniaturization is not trivial, and I'm concerned that most characterization plots will no longer apply once the ME antenna is made smaller. For instance, Fig 3(e,f) will be very different, and powering at large distances (>3cm) might no longer be possible. State-of-the-art ultra-small stimulating implants are much smaller (sub-mm) than the proposed technology that is currently in the mm scale.

Response: We greatly appreciate the feedback and concerns brought up in the two points above. The ME-BIT device presented here is significantly different from what was demonstrated by Singer et al. 2020. Our new device uses a custom integrated circuit (Fig. S6) that receives both power **and** digital data via the ME film. The device in Singer et al. 2020 was not programmable and the ME film was only used to receive power. Thus, this new device can be wirelessly programmed to change the stimulus amplitude and timing, which would be a critical feature for biomedical devices. We have made this point clearer by adding the following to line 99:

"Furthermore, in comparison to previous in-vivo demonstrations of ME-powered devices that were not digitally programmable [37], the ME-BIT technology described here can receive digital data via the ME effect to program the amplitude and timing of the electrical stimulus."

37. Singer, A. et al. Magnetoelectric Materials for Miniature, Wireless Neural Stimulation at Therapeutic Frequencies. Neuron 107, 631-643. (2020)

We have also added a more detailed discussion of how the power transfer will change as devices are made much smaller, which we include as the response to the next question.

Specific comments that the authors should address include:

• Line 30: This is the case for commercial devices but what about technologies developed in academic labs? The authors should mention the state-of-the-art sub-cm sized devices and novel minimally invasive surgical techniques (e.g., Cortese et al 2020, Khalifa et al 2021), mention their weaknesses, and explain the benefits of using the proposed technology. The reader might wonder: if the implant is very small and can be fully injected, then why use the vascular route which could lead to complications?

Response: Thank you for bringing up these questions along with relevant references. While our implant can be delivered through a minimally invasive procedure through a catheter, we acknowledge that it is not yet sub-mm scale and 'fully injectable'. The primary benefit of using the neurovascular approach allows for direct access to distal nerve targets without having to significantly displace any tissue for mm-sized implants, especially important if this technology were to be applied towards stimulating targets in the brain. Furthermore, to better contextualize our technology within the state-of-the-art, we updated the manuscript with the relevant discussion and references added in line 472:

"*As we miniaturize the implant and ME film sizes, we expect that the ME-BITs will be able to still function at centimeter depths in tissue. This is because the ME film voltage does not depend on the area of the film [37,43]. As a result, we expect that received power will only decrease linearly with the size of the film. The film voltage, on the other hand, is expected to remain constant, which will ensure that the voltages are large enough to operate the ASIC. Thus, we expect the major effect of miniaturization would be longer charging times between stimulation pulses, which could decrease the maximum stimulation bandwidth. Future work must also address packaging and monetarization, which will likely need to be changed as devices approach sub-mm length scales. These efforts will be needed to compare ME-powered implants with other types of sub-mm-sized battery-free implants and their compatibility with new minimally invasive delivery techniques, which are promising bioelectronic technologies but have yet to demonstrate neural stimulation in a large animal model [55-57]*"

55. Khalifa et al. A Simple Method for Implanting Free-Floating Microdevices into the Nervous Tissue. J. Neural Eng. 18 (2021)
56. Cortese et al. microscopic sensors using optical wireless integrated circuits. PNAS, 17, 9173-9179 (2020)
57. Lee et al. Neural recording and stimulation using wireless networks of microimplants. Nature Electronics, 604-614 (2021)

• Line 53 and 58: This depends on the size of the implant.

Response: We appreciate the comment and have qualified the sizes of implants that we expect to be able to use for minimally invasive procedures added in line 63:

"**Millimeter-sized** endovascular neural stimulators (EVNS) delivered via an intravascular catheter to deep tissue targets with a minimally invasive procedure through the blood vessels within the body would leave the tissue target undisturbed."

• Line 78: Not entirely sure this is true, please check the work by John Ho et al.

Response: Thank you for the feedback and recommendation. We originally assessed the work by John et al to have an antenna on the centimeter scale. The implant used for peripheral nerve stimulation in large animal models includes a meandering antenna that is 1.5 cm x 0.2 cm in comparison to our magnetoelectric transducers that are 5 mm x 1.75 mm. To more accurately distinguish our work, we have revised our claim in the manuscript on line 85:

*"There has yet to be a demonstration of a millimeter-sized wireless and **digitally programmable** neural stimulator that operates at a depth of several cm in a large animal model"*

• Line 90: Please add a reference.

Response: Thank you for the comment. We have added a relevant reference used in our comparisons to sub-mm ultrasonic implants on line 99.

29. Seo, D. et al. Wireless recording in the peripheral nervous system with ultrasonic neural dust, Neuron, vol. 91, pp. 529–539 (2016)
31. Piech, D. K. et al. A wireless millimeter-scale implantable neural stimulator with ultrasonically powered bidirectional communication. Nature Biomedical Engineering, vol. 4, pp. 207–222, (2020)
32. Sonmezoglu S. et al. Monitoring deep-tissue oxygenation with a millimeter-scale ultrasonic implant. Nature Biotechnology, 855-864 (2021)
33. Shi C. et al., Application of a sub-0.1-m3 implantable mote for in vivo real-time wireless temperature sensing, Science Advances, 7,19, (2021)

• Line 136: How was the surface coil designed? What specs were targeted?

Response: Thank you for the questions regarding the specs targeted when designing the coil, we primarily looked to designing coils that could allow for high translational and misalignment tolerances while still remaining in a form factor that could potentially be used as a wearable surface coil in the future. We have revised the manuscript to include some more details on our rationale for designing the surface coils in the transmitter methods section on line 576:

"We designed transmitter coils to provide uniform magnetic fields for characterization and provide a large alignment tolerance so that we could effectively power our devices in the operating room (OR). Based on COMSOL simulations we chose a spiral coil with an inner diameter of 6 cm with 15 turns and an outer diameter of 7 cm. We chose this size because it would be compatible with a wearable transmitter system [38] When the ME film is aligned parallel to the surface of the coil (as is the case for experiments in the OR) we place the coil off-center from the ME-BIT to power the device with the fringing fields. The impedance of the coil was measured to be ~ 0.5 Ω."

• Line 173 and 233: Please provide information on transmitted power and powering distance.

Response: Thank you for the feedback. For line 182, as long as the film can maintain the required operating voltage, the device can generate the estimated 4 mW of power which we have clarified in the text. Further characterization of the transmitter powers and distances are

added as suggested in line 259. Figure 3 is also updated with the new power transfer efficiencies in tissue. The revisions included are:

New Line 182: *"We estimate that this device can generate a maximum of 4 mW* ***as long as the ME films can maintain*** *a peak resonance voltage of > 8 Vpp with a resistive source impedance lower than 1 kΩ (Fig 2d)."*

New Line 259 for caption: "Measured power transfer efficiency for the magnetoelectric implant as a function of distance in tissue. At an operating distance of 30 mm, the transmitter power was ~ 6W to maintain the 1.17 mW implant power yielding a 0.01% efficiency"

• Line 246: Since the device is an implant, measured PTE should be done with tissue.

Response: Thank you for the feedback. We have conducted the power transfer efficiency experiment in an ex-vivo tissue model and updated figure 3. The figure changes include the new PTE graph and changing the table to a supplementary figure. We have also further clarified the methods and included supplementary figures used for calculating the PTE, including the rectified voltage charging curve of the implant.

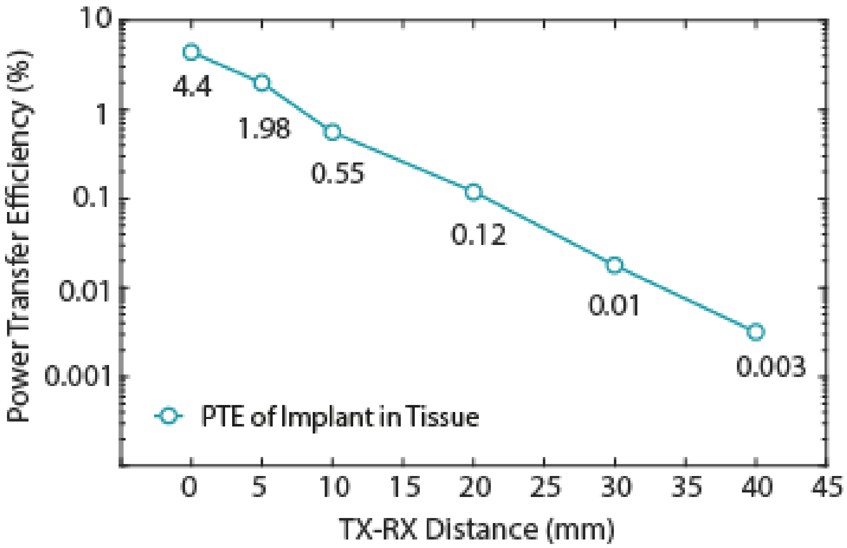

3f. Measured power transfer efficiency for the ME-BIT as a function of distance in tissue

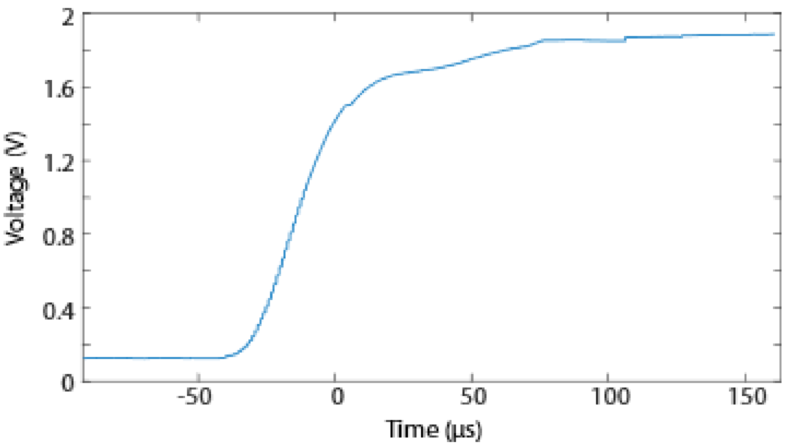

Supplemental Fig. 7 Charging curve to the minimum operating voltage of ~1.8V with measured rectified voltage of the ME-BIT.

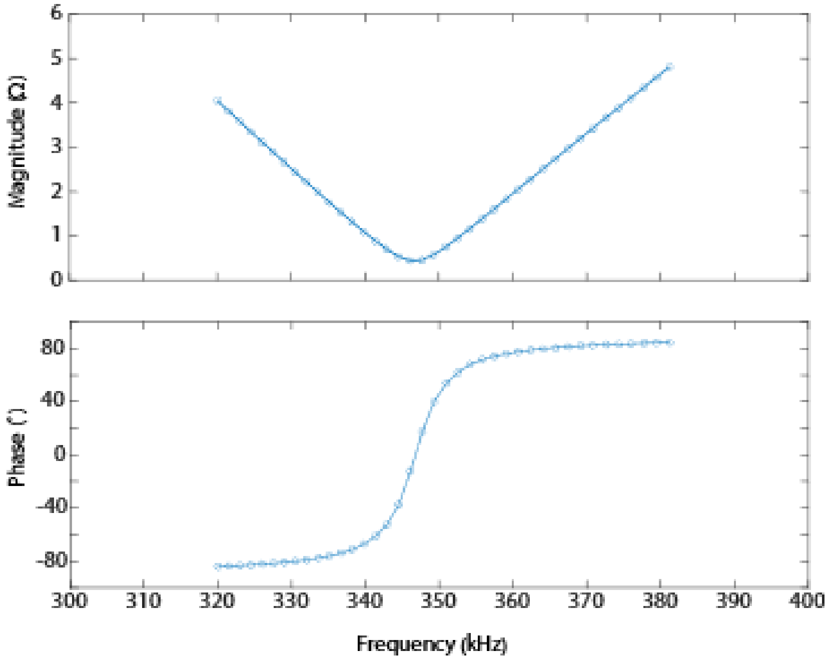

Supplemental Fig. 8 Impedance magnitude and phase of the resonant surface coil used to characterize power transfer efficiency. Resonant frequency of the transmitting coil is around 345 kHz

TX-RX Distance (mm)	Coil Current (A)	Peak Implant Power (mW)	Power Transfer Efficiency (%)
0	0.23	1.17	4.4
5	0.34	1.17	1.98
10	0.65	1.17	0.55
20	1.4	1.17	0.12
30	3.63	1.17	0.01
40	8.6	1.17	0.003

Table 1. Characterization of the power transfer efficiency for the ME-BIT as a function of distance. Input power to the implant was held constant while the coil current was increased as the distance between the transmitter and receiver increased.

On line: 312*: "At the surface of the coil, while the ME-BIT generated a peak power of 1.17 mW, the resulting peak efficiency of the implant was found to be 4.4% (Fig. 3f). In order to maintain a functional voltage on the implant at a depth of 4 cm, the coil current was increased from 0.23 A to 8.6 A at 0 mm and 40 mm distance respectively (Table 1)."*

• Line 256: Since the ME antenna is the main novelty it would be interesting to discuss how it compares to other miniaturized implants that utilize ME antennas in line 265.

Response: We appreciate the comment and have included some discussion on comparing our work using ME to other existing implants using ME antennas in line 291.

In line 291: "*Existing devices and implants that use ME antennas likely share similar angular tolerances and have been shown to be operational at large distances; however, these sub-millimeter devices primarily operate at much higher frequencies (60 MHz to 2.5 GHz) [45-47]. At these higher frequencies, tissue absorption and reflection become more significant, which lowers the amplitude of the field that can be applied within the safety limits [48]. Furthermore, many of these demonstrations rely on the magnetic component of radiating electromagnetic waves, which is small compared to the electric field component. As a result, small ME devices that couple to radiating electromagnetic waves are used primarily for low power sensing and communication applications rather than electrical stimulation, which requires more power."*

45. Nan et al. Acoustically actuated ultra-compact NEMS magnetoelectric antennas, Nature Communications, 8: 296 (2017)
46. Zaeimbashi M. et al. NanoNeuroRFID: A wireless implantable device based on magnetoelectric antennas, IEEE Journal of Electromagnetics, RF and Microwaves in Medicine and Biology, 3,3, pp. 206-215 (2019)
47. Zaeimbashi M. et al., Ultra-compact dual-band smart NEMS magnetoelectric antennas for simultaneous wireless energy harvesting and magnetic field sensing. Nature Communications, 12, 3141 (2021)

48. IEEE Standard C95.1-2019 2019 IEEE standard for safety levels with respect to human exposure to electric, magnetic, and electromagnetic fields, 0 Hz to 300 GHz

• Line 296: Details about the electrode should be provided.

Response: Thank you for the comment. We have added the following description of the stimulating electrodes into the figure caption in line 323:

"The stimulating electrodes are two 1 mm x 1 mm Au pads spaced 2 mm apart on the bottom side of the circuit board."

• Line 392: The strength of the research lies in this experiment. However, since the implant is bulky the surgery is not minimally invasive which makes the concept less appealing.

Response: The implant can be deployed through a 9 Fr sheath (inner diameter of 3.09 mm) meaning that it can be delivered via a minimally invasive percutaneous procedure, which we demonstrate in Fig. 5f. The open surgery as shown in Fig. 5d is performed so we can visualize the location of the ME-BIT.

We have updated the text to on line 418 to emphasize this point:

"Because the implant can be deployed through a 9 Fr sheath (inner diameter of 3.09 mm), it would be possible to deliver the device using minimally invasive surgical procedures. For example, Fig. 5f shows an X-ray image of an endovascularly deployed ME-BIT in the femoral artery (see methods)."

We have also updated the Fig. 5f caption to read: "*An X-ray image of the ME implant endovascularly deployed from a 9 Fr sheath into the femoral artery. The ME film, capacitor, and SoC can be seen in X-ray."*

• Line 411: This is the biggest weakness of the research. Although the ME antenna shows multiple benefits over other types of powering methods it does not mean the device has "excellent scaling properties". There are multiple challenges of scaling an implant, for instance, new packaging techniques will need to be applied, the operating distance will be significantly reduced, the techniques used to make the ME antenna will change as it might have to be microfabricated, the connection between the IC and the electrode will be more challenging, etc. The authors should address some of these challenges instead of simply showing the very high PTE of a relatively large ME antenna.

Response: We appreciate the feedback. To clarify this statement, we have revised the text on line 437 to read:

"Furthermore, because wireless power transfer scales favorably for ME in that power decreases linearly with implant size rather than a higher power as is the case with other wireless power technologies [37] ...."

We have also added the previously mentioned discussion of the additional challenges facing scaling to smaller sizes to the discussion on line 472:

“As we miniaturize the implant and ME film sizes, we expect that the ME-BITs will be able to still function at centimeter depths in tissue. This is because the ME film voltage does not depend on the area of the film [37,43]. As a result, we expect that received power will only decrease linearly with the size of the film. The film voltage, on the other hand, is expected to remain constant, which will ensure that the voltages are large enough to operate the ASIC. Thus, we expect the major effect of miniaturization would be longer charging times between stimulation pulses, which could decrease the maximum stimulation bandwidth. Future work must also address packaging and connectorization, which will likely need to be changed as devices approach sub-mm length scales. These efforts will be needed to compare ME-powered implants with other types of sub-mm-sized battery-free implants and their compatibility with new minimally invasive delivery techniques, which are promising bioelectronic technologies but have yet to demonstrate neural stimulation in a large animal model [55-57]”

55. Khalifa et al. A Simple Method for Implanting Free-Floating Microdevices into the Nervous Tissue. J. Neural Eng. 18 (2021)
56. Cortese et al. microscopic sensors using optical wireless integrated circuits. PNAS, 17, 9173-9179 (2020)
57. Lee et al. Neural recording and stimulation using wireless networks of microimplants. Nature Electronics, 604-614 (2021)

Reviewer #2 (Report for the authors (Required)):

* A brief summary of the results:
Magnetoelectrics hold promise to enable miniaturization of electronics for blood vessels.
Fabricated an ASIC encapsulated in a 3 x 2.15 x 14.8 mm package.
A custom magnetic field transmitter was used – at up to 4cm depths.
Can generate up to 4 mW of >8 Vpp.
ASIC that uses the digitally received data to program the shape (mono-phasic or bi-phasic), the amplitude (0.3 V to 3.3 V with 4-bit resolution), the pulse width (0.05 ms to 1.2 ms with 3-bit resolution), and the delay (0.01 ms to 0.8 ms) of the stimulation.
The ASIC, fabricated on 180 nm complementary metal-oxide-semiconductor (CMOS) technology (TSMC), measures only 1 by 0.8 mm.
ME-BITs can tolerate approximately 3 cm misalignment from the center of the transmitter coil and a depth of 3 cm in tissue.
Deployed our ME-BIT through an 9Fr sheath into the femoral artery.
ME-BIT can be implanted deep within the tissue close to targeted areas without requiring lead wires that connect to a more superficial inductive coil.
First example of a magnetoelectric-powered bioelectronic implant in a large animal model.

* Your reasoned opinion on the degree of advance (fundamental, mechanistic, methodological, technological, therapeutic, translational and/or clinical) of the work with respect to the state of the art. If the results or conclusions are not original, please provide relevant references:

This work represents a major technological advancement that solves a clinical problem. More work needs to be done to answer questions regarding viability for clinical delivery but the foundational science and validation data are extremely encouraging.

* Your reasoned opinion on the broad implications of the findings:

Solving the problems of wireless power delivery to enable minimally invasive / endovascular stimulation could create a new industry. This solution, although early, shows potential as a significant breakthrough.

* Any major technical criticisms or questions.
none

* Any minor technical criticisms or questions.
None

Response to reviewer: Thank you for the review. We have included the following discussion related to the limitations and potential future work necessary for chronic implantation and translation such as miniaturization and biocompatibility in the discussion along with some new relevant references:

In line 455: *"Future studies are needed to determine how chronic deployment of the ME-BIT within the blood vessel could affect vasculature health as well as the biocompatibility of the device, including if a hermetically sealed capsule is suitable for long term implantation of the lead-containing PZT or if there are suitable piezoelectric alternatives, such as PVDF that do not contain lead [37]."*

In line 472: "*As we miniaturize the implant and ME film sizes, we expect that the ME-BITs will be able to still function at centimeter depths in tissue. This is because the ME film voltage does not depend on the area of the film [37,43]. As a result, we expect that received power will only decrease linearly with the size of the film. The film voltage, on the other hand, is expected to remain constant, which will ensure that the voltages are large enough to operate the ASIC. Thus, we expect the major effect of miniaturization would be longer charging times between stimulation pulses, which could decrease the maximum stimulation bandwidth. Future work must also address packaging and connectorization, which will likely need to be changed as devices approach sub-mm length scales. These efforts will be needed to compare ME-powered implants with other types of sub-mm-sized battery-free implants and their compatibility with new minimally invasive delivery techniques, which are promising bioelectronic technologies but have yet to demonstrate neural stimulation in a large animal model [55-57]"*

55. Khalifa et al. A Simple Method for Implanting Free-Floating Microdevices into the Nervous Tissue. J. Neural Eng. 18 (2021)
56. Cortese et al. microscopic sensors using optical wireless integrated circuits. PNAS, 17, 9173-9179 (2020)
57. Lee et al. Neural recording and stimulation using wireless networks of microimplants. Nature Electronics, 604-614 (2021)

Reviewer #3 (Report for the authors (Required)):

The paper reports on a mm-scale neural stimulation implant that is powered wirelessly. The wireless power receiver is a magnetoelectric laminate (Metglas-PZT). The implant's size is such that it could feasibly, with more engineering, be implanted inside a blood vessel and be delivered endovascularly. The implant was demonstrated on both small and large animal models.

The manuscript is well-written and well-organized. The system is technologically impressive and the animal model demonstrations are compelling. 4 mW of power at a receiver of size 0.3 X 1.75 X 5 mm is very impressive and compares well with the state of the art. This is a high quality manuscript that I believe will be of interest to readers and demonstrates significant technological advancements to the state of the art.

I have a few minor questions and recommendations as described below.

1. As PZT has lead, it might be worthwhile to address the safety of PZT implants.

Response: We appreciate the comment and agree that PZT is not a biocompatible implantable material on its own. However, we think that future studies are warranted to determine if a hermetically sealed package will allow for the use of PZT. Alternatively, other biocompatible piezoelectric materials can be explored to be used as a replacement for PZT. We have updated the manuscript to include this discussion on line 455:

"Future studies are needed to determine how chronic deployment of the ME-BIT within the blood vessel could affect vasculature health as well as the biocompatibility of the device, including if a hermetically sealed capsule is suitable for long term implantation of the lead-containing PZT or if there are suitable piezoelectric alternatives, such as PVDF that do not contain lead. [37]"

37. Singer, A. et al. Magnetoelectric Materials for Miniature, Wireless Neural Stimulation at Therapeutic Frequencies. Neuron 107, 631-643. (2020)

2. 1 mT at ~ 345 kHz is a large field and exceeds both IEEE and ICNIRP safety standards. (These standards have been recently updated; safe fields at this frequency have been increased, but not up to 1 mT.) The authors should at least address the safety issues of transmitting fields > 1 mT at these frequencies into the human body.

Response: We thank the reviewer for raising our awareness to ICNIRP standard and have added a discussion of the how these safety limits apply to our device on line 459:

"Another safety concern for the long-term implantation of the ME-BIT is the interactions between the applied magnetic field and biological tissue. Our COMSOL simulations show that a field of 1 mT at an implant depth of 3 cm corresponds to a surface magnetic field of 7.7 mT, which results in an electric field and SAR that are within the IEEE safety limit of 101 V/m and 2 W/kg for unrestricted environments [36]. For other guidelines such as by ICNIRP that have lower limits for magnetic field exposure, this device operates outside the compliance range [54]. Thus, future approval for these devices may depend on which standards are applied by the regulatory body. While we operate our device at an optimal rectified voltage of 2.5 V, the ME-BIT remains operational at voltages as low as 1.8 V, in which field strengths as low as 0.6–0.8 mT can still be used. Additionally, improvements to the ME materials that increase the PTE or reduced power consumption by the ASIC could allow these devices to operate with lower magnetic field strengths, which could make the devices compliant with additional safety standards."

36. Alrashdan et al. Wearable Wireless Power Systems for ‘ME-BIT’ Magnetoelectric-Powered Bio Implants. Journal of Neural Engineering 18, 4. (2021)
54. ICNIRP. Guidelines for limiting exposure to electromagnetic fields (100 kHz to 300 GHz). Health Phys 118(00):000-000 (2020)

3. What specific Metglas formulation is used? Is the Metglas prepared in any way (i.e. annealed, pre-magnetized)?

Response: Thank you for the question regarding the Metglas formulation.

We have included the specific Metglas alloy in the manuscript on line 500:

*“The magnetoelectric film is fabricated with a 127 um thick PZT (APC Int.) bonded to a 23 um thick layer of **unannealed** Metglas **2605SA1** (Metglas Inc.) with a thin epoxy layer (Hardman Double/Bubble).”*

4. The vibration mode of the ME device is not clear. Given the size and frequency, I assume an extensional vibration mode. There would be other modes, such as a primary bending mode at different frequencies. Did the authors do a full frequency sweep of the response of the ME device? Figure 2 only shows 250 – 450 kHz. Why choose this frequency range?

Response: Thank you for the feedback. We are indeed operating in the extensional vibration mode. We have observed the primary bending mode at lower frequencies, but it has been shown that operating ME bilayers at that resonance mode yields slightly lower voltage coefficients. As we further optimize our ASIC and transmitter designs, we may not need as high voltages and will look to use other resonance modes. We have clarified the vibration mode that we are using in the text and added some rational in the text:

Caption on line 194: *“The peak-to-peak voltage for a film resonating in the fundamental **extensional vibration mode** at 345 kHz as a function of magnetic field frequency.”*

In line 501: *“The films are then laser cut by a femtosecond laser cutter to the desired shape to operate in the 300-400 kHz. At this frequency range, the ME films operate in the fundamental extensional vibration mode. We chose to operate the ME-BIT in the extensional vibration mode as it has been shown previously with ME bilayer laminates that while the bending mode has higher magnetoelectric energy conversion efficiency, longitudinal resonance modes yield slightly higher voltage coefficients [60]. Future work can consider the usage of different resonant modes including the primary bending mode at lower resonant frequencies.”*

60. Wan, J. et al., Strong flexural resonant magnetoelectric effect in Terfenol-D/epoxy-Pb(Zr,Ti)O3 bilayer, Appl. Phys. Lett. 86, 202504 (2005)

5. How is the device, and specifically the ME film, encapsulated for the experiment in the large animal model? It looks like the ME film is directly encapsulated in the epoxy without any air gap. If this is the case, I suspect that the epoxy is significantly dampening the vibrations of the film. Better performance might be obtained by encapsulating the ME film so that it can vibrate more freely.

Response: Thank you for the question. For the endovascular implant, it is assembled with the ME film before being placed within the 3D printed capsule which allows for an air gap around

the film. The entire capsule is then coated with non-conductive epoxy. We have clarified this in the methods section for fabrication the ME-BIT on line 521:

"The assembled device is then placed within a 3D printed air-filled PLA capsule which allows the film to freely vibrate in air. The entire capsule is then sealed with non-conductive epoxy to provide more structural stability and prevent moisture from infiltrating the device. The assembled implant's final dimensions are 3 x 2.15 x 14.8 mm."

6. The text on the figures (e.g. Fig. 2) is very small and hard to read.

Response: Thank you for the feedback on the figures. The text sizes have been increased for readability.

7. On line 207, the definition of stimulation efficiency (n_stim = stimulation amplitude / stimulation supply) is a little odd to me. Perhaps this is a standard definition for implant stimulation, but efficiency is typically defined with power or energy ratios, not voltage ratios. A high stimulation efficiency, defined as voltage ratios, does not necessarily imply that heating won't be an issue.

***Response**: We appreciate the feedback on the stimulation efficiency. We have updated the manuscript to include the current term in the equation for n_stim as well as clarified the description for the benefits of having a high stimulation efficiency in the ASIC on line 217:*

"...; when compared to the stimulation power (< 9 mW), the power consumption of the SoC is negligible (<9 uW). Thus, we expect little heating due to energy loss on the chip. Furthermore, this high efficiency also reduces required transmitter power and its associated heating"

$$\eta_{stim} = \frac{< stimulation\ voltage > \times < stimulation\ current >}{< stimulation\ supply\ voltage > \times < stimulation\ current >}$$

8. The authors demonstrate via simulation and experimentation that the implant is robust to 3 cm of lateral misalignment. That is good. However, their arguments about angular misalignment are weaker. They simply claim better angular misalignment than other mm-scale technologies by referring to another study. I would suggest that the authors either leave out the paragraph about angular misalignment (lines 253 – 259), which is not really critical to the paper anyway, or provide quantitative simulation based angular misalignment performance.

Response: We appreciate the feedback and great suggestions on better quantifying the angular misalignment performance. We have built a COMSOL simulation which includes skin, fat, and muscle layers along with our encapsulated implant inside the tissue. Using this model, we computed the angular misalignment tolerance of the implant in two degrees of rotation, theta and phi. Furthermore, to replicate our in-vivo experiment where the device is completely horizontal with respect to the surface of the coil, we can offset the coil so that the horizontal implant is closer to the edge of the coil and can still be successfully powered at centimeter distances. We have included the simulation results of angular misalignment in-vivo in our main figure 3, included a description of the methods, and updated the caption as follows:

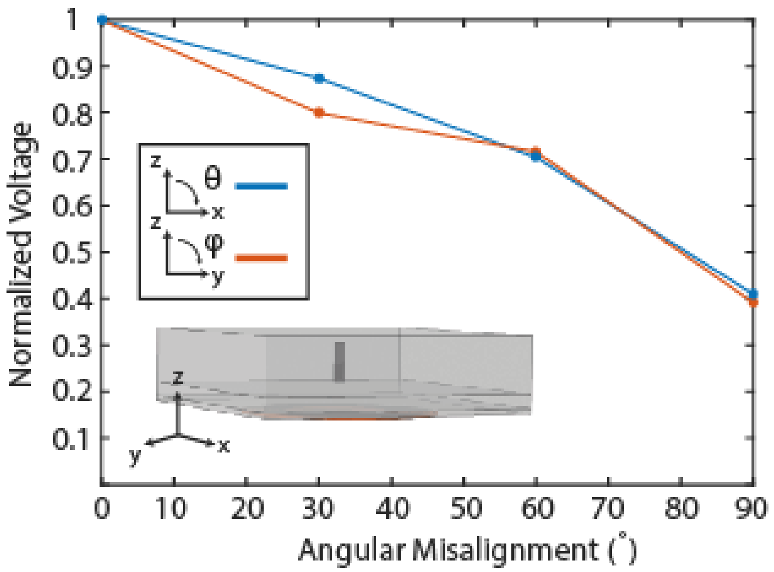

Fig 3d. COMSOL simulation of the ME-BIT placed within a layered block of tissue (20 mm muscle, 5 mm fat, 2 mm skin) is used to model angular misalignment tolerances in-vivo for both θ and φ angular rotations.

The main text was also updated to include a discussion of the results in line 285:

"To assess the angular tolerance of the ME-BIT in vivo, we used a COMSOL model to simulate how ME voltage is affected when it undergoes angular misalignment in tissue (see methods). Because the simulated coil is radially symmetrical, we found that rotating the film in either the θ direction, as shown in Fig. 3d, or in the φ angular direction, resulted in the ME voltage decaying similarly with either angular change and being able to maintain > 40% of the maximum voltage at a 90° rotation."

We also included an updated description of the methods describing this new experiment in line 589:

"The COMSOL model for the angular misalignment analysis used a similarly sized 7 cm diameter coil. A 5 mm x 1.75 mm x 0.023 mm Metglas sheet is placed within a 14 mm x 3 mm x 2.15 mm airbox to simulate the ME-BIT. To model how the device would behave in vivo, the ME-BIT is placed within a tissue layer model (20 mm of muscle, 5 mm of fat, and 2 mm of skin) at the distance of 15 mm consistent with the large animal experiments. The device is then rotated in two different directions (θ, φ). For each angle, the transmitter is translated to the position that achieves peak voltage across the film. For example, at a 90° rotation, the ME-BIT is placed off-center to maximize the use of the fringing fields. Because we operate at the linear region of the magnetostrictive curve, strain induced on the magnetostrictive layer is linearly related to the induced voltage on the ME film. [38, 59] Thus, we use the simulated strain induced in the Metglas film to calculate the induced voltage and normalize this voltage to the peak value. (Fig. 3d)"

9. Line 419. I'm not so sure that magnetic fields at 350 kHz will easily penetrate metal casings. Glass and ceramic should be fine.

Response: Thank you for catching this oversight. We have removed metal casings in the sentence and updated the manuscript in line 447:

"While thin film packaging solutions have yet to be fully developed for clinical use, other wireless implants have shown that glass or ceramic casings can enable chronic operation [43]. Fortunately, the magnetic fields should easily penetrate these materials and thus they are not expected to degrade the power coupling efficiency."
